# Tracing spatiotemporal changes in agricultural and non-agricultural trade networks of India

**DOI:** 10.1371/journal.pone.0286725

**Published:** 2023-09-26

**Authors:** Sujata Kulkarni, Raviraj Dave, Udit Bhatia, Rohini Kumar

**Affiliations:** 1 Discipline of Civil Engineering, Indian Institute of Technology Gandhinagar, Palaj, Gandhinagar, Gujarat, India; 2 Dr. Kiran C. Patel Center of Sustainable Development, Indian Institute of Technology Gandhinagar, Palaj, Gandhinagar, Gujarat, India; 3 Computational Hydrosystems, Helmholtz Centre for Environmental Research, UFZ, Leipzig, Germany; Balochistan University of Information Technology Engineering and Management Sciences, PAKISTAN

## Abstract

The evolving international economic instability and international trade relationship demand a nation to move towards a self-reliant integrated system at a sub-national scale to address the growing human needs. Given India’s role in the global trade network, it is critical to explore the underlying extensive complex trade network at the domestic scale. The potential advantages of complex interaction among the different commodities remain unexplored despite the known importance of trade networks in maintaining food security and industrial sustainability. Here we perform a comprehensive analysis of agricultural flows in contrast with non-agricultural commodities across Indian states. The spatio-temporal evolution of the networks from 2010–2018 was studied by evaluating topological network characteristics of consistent spatially disaggregated trade data. Our results show an increase in average annual trade value by 23.3% and 15.4% for agriculture and non-agriculture commodities, respectively, with no significant increase in connectivity observed in both networks. However, they depict contrasting behavior concerning the spatio-temporal changes, with non-agriculture trade becoming more dependent on production hubs and the agriculture trade progressing toward self-reliance, which signifies the evolution of the diversification in the existing agrarian trade network. Our findings could serve as an important element in deepening the knowledge of practical applications like resilience and recovery by devising design appropriate policy interventions for sustainable development.

## Introduction

Rapid acceleration in urbanization and intensive population growth has elevated the global and regional demand for commodities, making the trade network vital to cope with the changing market [1, 2]. The recent advances in the connectivity of transportation infrastructure with the new trade policies have facilitated the growth of the trade network and made it more interconnected globally [3, 4]. While the international trade network provides economic leverage to participating nations, it also poses an economic and financial threat to the highly interconnected countries during economic shock due to external stresses, such as natural disasters, wars, and global pandemics [5–9]. The few cases of such a disruption to the international trade network are the Thailand flood (2011) [6], Japan earthquake and nuclear disaster (2011) [6, 10], Hurricane Harvey in the United States (2017) [11], and the COVID-19 pandemic (2019) [12]. Moreover, the sensitivity of international trade to exchange rates [13], transportation costs and delay [14], culture [15], and geopolitics [16] makes the network fragile, which can imperil a nation’s food security and impair its economic condition. The fragility of the international trade network highlights the importance of a localized (interstate) trade network within the country, which can reduce the shock and minimize the damage, especially in developing nations.

Hence it is crucial to map and quantify the spatio-temporal changes of interstate trade to understand the robust characteristics of such systems. Moreover, such analysis is pertinent to understand the temporal shifts in commodity hubs: states/nodes, which are key importers/exporters, as these nodes will play a disproportional role in governing flows across the network. The interaction of resources within a nation outlines the characteristic of the domestic trade networks of the country, enabling us to gain insights into a localized trade network. The complex network approach has garnered considerable attention from the scientific community in understanding networks’ structural and dynamic behavior in disparate domains and scales [17, 18]. In the case of a trade network, the complex network analysis help in quantifying underlying properties, which in turn helps in untangling and uncovering its underlying characteristics [2, 19]. Previous work on the trade networks has mainly focused on global networks [2, 17, 19–25] or the interaction of a single commodity among many nations [2, 26–31]. The literature has produced analytical network topology based research on food flow network at various spatial scale with consideration of multiple commodities including cereal crops, cash crops, and live-stocks [32–34]. However, comparative analysis of multiple agricultural commodities and non-agricultural commodities using network analysis has not been explored.

Studies on the domestic exchange of resources and its changes over time periods in trade networks seldom exist, especially in developing countries. The significant challenge in quantifying and analyzing the domestic trade network in developing nations is the paucity of observational data on the trade network. Understanding the intertwined domestic trade networks with a complex network approach helps to identify the hubs in the supply chain system, which, if perturbed, can disrupt the entire supply chain [19, 33, 35, 36]. Analyzing the evolution of trade networks is crucial to capture the uncertainty and fluctuations associated with trading volumes over temporal and spatial scales. Further, it also helps in analyzing the resilience and recovery of the network to understand the responses to policy interventions [36–39].

In this study, We use a complex network lens on interstate trade networks to address the following questions: (1) How has the trade of agricultural and non-agricultural commodities changed spatiotemporally across India? (2) What are the key differences in network properties of agricultural and non-agricultural commodity transfer networks? and (3) Do leading exporters also lead imports of commodities in interstate trade networks, and how have such patterns evolved over a recent period for agricultural and non-agricultural commodities? To answer these questions, we analyze the changes in network properties of the Domestic Indian Trade Network (DITN) for agricultural and non-agricultural commodities (explained in the Method section). We quantify the network’s topological features to understand the trade network’s structural characteristics over the temporal window of 2010 to 2018. We examine the role of different states in trade and their contribution to the import and export of these two types of commodities corresponding to agricultural and non-agricultural trades. Finally, to quantify the relationship between import and export in agriculture and the non-agriculture DITN, we analyze the inward and outward movement of commodities over the Pan-India spatial scale. We also explore the relationship between trade import/export and non-topological parameters, including population and Gross State Domestic Product (GSDP) at Purchasing Power Parity (PPP). Our study offers new insights into understanding the interaction of commodities over a regional scale and helps us identify the evolution of hotspots of the trade network, which can further aid in improving trade policies and identifying the network’s Achilles heels.

We organize the rest of the manuscript as follows: In Section 2, we first discuss the details of the study area and data sets used in the study. Then, we present the analysis overview, the construction of DITN, and the topological characteristics of a network. After this, we brief the changes in the trade network spatiotemporally and compare classes of commodities. Section 3 demonstrates the results. Finally, in Section 4, we discuss the spatio-temporal changes in interstate trade networks.

## Materials and methods

### Data and network construction

We obtain the interstate movement of resources data for 2010–2018 from the Directorate General of Commercial Intelligence Statistics (DGCI&S), Government of India [40]. The DGCI&S database has trade data of agricultural and non-agricultural commodities transferred through Indian railroad networks, which moves 80% of the stocks in India [41, 42]. The traded flow of resources between two states in raw data is given in quintals (1 quintal = 100 kg).

We convert the trade data volume into equivalent trade value (Indian rupees; ₹) through governmental data sets on interstate trading prices [43–51]. Here we use the annual minimum support price rate of different agrarian commodities, while for industrial products, we consider the annual retail price rate for each year from 2010–2018 (Table S1 in S1 File). This monetary translation helps to remove the bias towards the bulkier commodities transported through the network while comparing agricultural and non-agricultural commodities. Here, we use harmonized commodities data. Harmonized system codes for commodities are typically used in the export process for goods to classify the traded products [52]—and classify the commodities into two main categories: agriculture and non-agriculture, to understand the aggregated level trade transfer. Categorizing commodity flow data as agriculture and non-agriculture commodities enables us to distinguish the unique aspects of commodity flow across the space and time. (Table 1) shows the sorting of different commodities into two categories.

**Table 1:** Classification of commodities.

Category	Commodity
Agriculture	rice, gram, and gram products, pulses, wheat and wheat products, jowar, bajra, oilseeds, fruits, vegetables, fodder and husk, jute, coffee, spices.
Non-Agriculture	metal ores, coal, coke, cement, iron steel, fertilizers, marble stones, limestone, dolomite, gypsum, kerosene, sugar.

**Fig 1:**
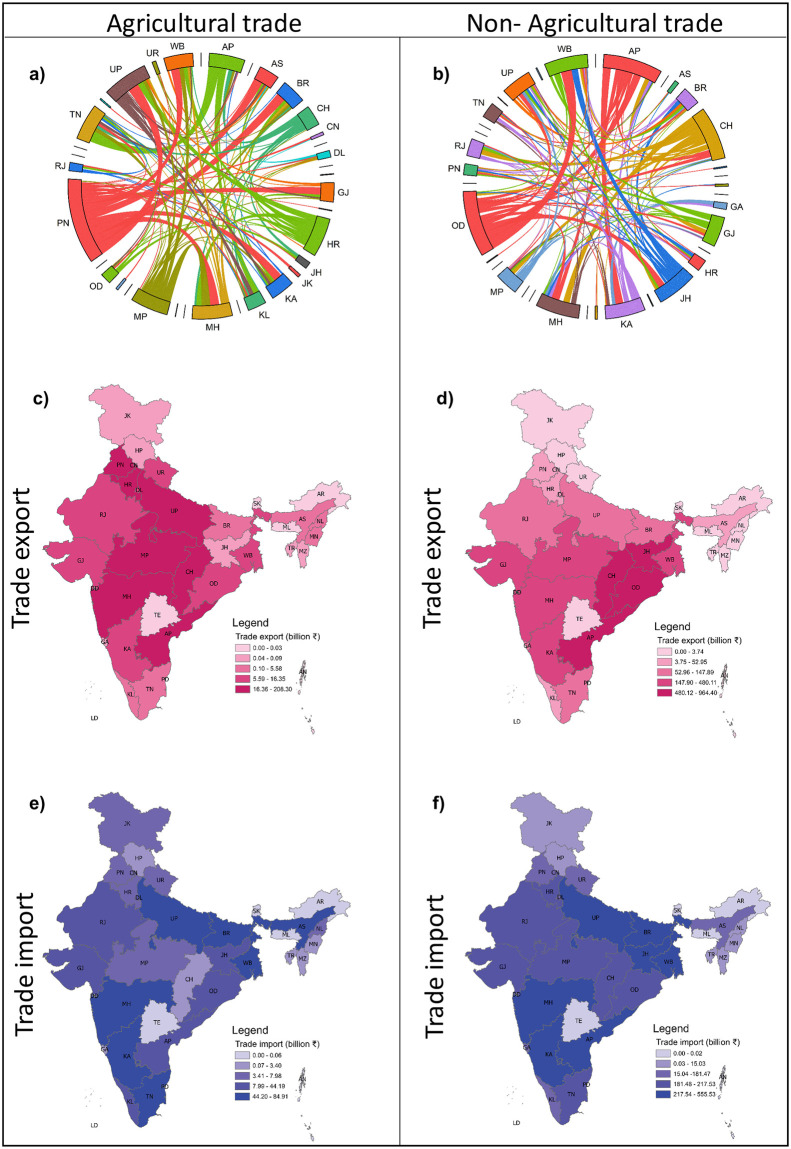
Interstate trade of agriculture and non-agriculture commodities in India. (a) and (b) show the chord diagram representing the time-averaged agricultural exports and non-agricultural commodities, respectively. The link indicates the flow between different states, the link width showcases the trade volume in ₹, and the links’ color corresponds to the exporting regions. (c) and (e) shows time-averaged exports and imports for agriculture, whereas (d) and (f) depict the same for non-agriculture commodities. The state is represented by two letters. All averages are calculated for the period 2010–2018.

We aggregate the harmonized commodity-specific trade matrices to obtain the total annual agricultural and non-agricultural trade volume across the states for nine consecutive years (2010–2018). The data summarize the resource flows between states and can be represented as a weighted directed network. In the network, the node *i* represents the state in India. The link *l*_*ij*_ demonstrates the flow of resources between states *i* and *j*. The direction of flow (i → j) indicates the relationship between the exporter (*i*) and importer (*j*). The elements of adjacency matrix (*A*_*ij*_) = 1 if there is trading between two states. After the construction of links, weight *w*_*ij*_ (trade value) is attributed to each link. In this study, the monetary value of trade (₹) is used as a weight to represent the flow between the two states. Thus we construct the weighted directed trade networks each year for *N* = 36 nodes, representing 28 Indian states and 8 Union Territories (UT) with pre-defined political boundaries (Fig 1c). We analyze the DITN primarily through two aspects: the topology of the trade network and the spatio-temporal characteristics of the DITN.

### Topological characteristics of network

We consider multiple metrics to characterize the topological characteristics of the network that helps to analyze the overall structural characteristics of the agriculture and non-agriculture DITN over the temporal window of 2010–2018.

#### Degree of node

The degree of a node (*k*) [53] represents the total number of links connected to a given node. Since DITN is a directed network, it has two degrees associated with each node, in-degree (state from which it imports) and out-degree (states from which commodities are exported). We evaluate the network’s average degree, which depicts the average number of links per node. The network’s average in-degree and average out-degree for directed interstate trade networks are given by (Eq 1):

$$\begin{array}{c} <k_{i}^{in}>=\frac{1}{n}\sum_{i=1}^{n}k_{i}^{in}=<k_{i}^{out}>=\frac{1}{n}\sum_{i=1}^{n}k_{i}^{out} \end{array}$$ (1)

where, $<k_{i}^{in}>$ and $<k_{i}^{out}>$ denote the average in-degree and average out-degree, respectively. The $<k_{i}^{in}>$ and $<k_{i}^{out}>$, for example, represent the bulk export and import of the agriculture and non-agriculture interstate trade network.

#### Network density

Network density (*D*) in DITN indicates connectedness among the trading partners of a network. In DITN *G*, with *N* number of states and *L* possible trade connection between the states, the density of the DITN is calculated using (Eq 2) [54]. A higher network density depicts a larger proportion of trading partners connected in the DITN.

$$\begin{array}{c} D=\frac{L}{N(N-1)} \end{array}$$ (2)

where, *N*(*N* − 1) is the number of maximum possible connections in a network of size *N*.

#### Clustering coefficient

The clustering coefficient of DITN is an indicator of the degree of connectedness among the neighboring states (here, neighbors do not refer to geographical neighbors. In this context, two states are considered to be neighbors, if there is direct commodity exchange between them). If a network exhibits a large clustering coefficient, it indicates that multiple alternate connections exist among the neighbors of the state under consideration. Hence, even if the trade activities of a particular state are disrupted for any reason, the neighbors don’t get isolated from the rest of the network. The clustering coefficient in the directed network is calculated using (Eq 3).

$$\begin{array}{c} C_{i}=\frac{L_{i}}{k_{i}\left(k_{i}-1\right)} \end{array}$$ (3)

where, the *C*_*i*_ is the clustering coefficient of *i*^*th*^ node with value between 0 and 1, *L*_*i*_ is the number of links between the *k*_*i*_ neighbors of node *i*.

The degree of clustering for the whole network is captured using the average clustering coefficient < *C* >. The < *C* > is calculated as:

$$\begin{array}{c} <C>=\frac{1}{n}\sum_{i=1}^{n}C_{i} \end{array}$$ (4)

Higher order of topological network properties examines the neighborhood properties and nodes’ importance in the network.

#### Betweenness centrality

It represents the state’s importance through the measure of the extent to which a given state lies on paths between the other states in DITN. The measure considers the shortest path between each state pair in trade. Mathematically it is given by (Eq 5).

$$\begin{array}{c} C_{Bi}=\sum_{s,t\in V}\frac{\sigma_{st}\left(i\right)}{\sigma_{st}} \end{array}$$ (5)

where C_*Bi*_ is the betweenness centrality of node i, V is the set of nodes in the network, *σ*_*st*_ is the number of shortest paths from node s to node t, and *σ*_*st*_(*i*) is the number of those shortest paths that pass through node i.

Here we consider the longitudinal analysis of the trade network through a centrality measure of the entire network and how it evolves. This allows us to understand the potential influence and temporal nature of states’ trading interaction in agricultural and non-agricultural DITN.

#### Modularity

It is a measure to evaluate the extent to which the links in the network are clustered into groups of communities [55]. The modularity indicates the degree to which the DITN can be divided into relatively independent groups of trading states. These trading groups of states are characterized by a high level of trade connection within groups and a low level of connectivity between the groups (Eq 6).

$$\begin{array}{c} Q=\frac{1}{2m}\sum_{i,j}[A_{ij}-\frac{k_{i}k_{j}}{2m}]\delta \left(c_{i},c_{j}\right) \end{array}$$ (6)

where Q is the modularity of the network, A_*ij*_ is the element in the adjacency matrix at row i and column j, k_*i*_ is the degree of node i, m is the number of edges in the network, c_*i*_ is the community membership of node i, and *δ*(*c*_*i*_, *c*_*j*_) is the Kronecker delta function, which is equal to 1 if c_*i*_ = c_*j*_ and 0 otherwise. Here we consider the Louvain algorithm for maximizing the modularity [56].

### Spatial and temporal variations in trade and network related measures

We evaluate the spatial variations in trends for the export and import of agriculture and non-agriculture DITN. We use linear regression for a trend line analysis with a 95% confidence interval. We check the statistical significance of the trends using the Mann-Kendall test. To evaluate the relative importance of nodes within the network, we analyze three centrality measures: strength (a measure of weighted connectivity), betweenness (a measure of states’ importance with respect to the flow of trade in the network), and PageRank (a measure of importance due to connection with highly influential states in the network) [57]. In addition to connectivity, the volume/value of traded commodities within the trade network should be considered to account for the relative strengths of these connections. Hence, we construct equivalent weighted networks for all years. In the weighted directed network, we use the in-strength and out-strength of a node to identify the amount (₹) of imports and exports across the interstate trade network (Eq 7).

$$\begin{array}{c} s_{i}^{in}=\sum_{j=1}^{n}A_{ji}w_{ji}\;,\;s_{i}^{out}=\sum_{j=1}^{n}A_{ij}w_{ij} \end{array}$$ (7)

where, $s_{i}^{in}$ and $s_{i}^{out}$ denote the in-strength and out-strength of a state, and *w*_*ij*_ is the traded value of commodity from node *i* to node *j*. Strength centrality is then computed through the sum of the weights of in-degree and the weights out-degree of a node.

We use PageRank [57] to estimate the influence of the state with respect to the transfer of commodity by assigning a score to each node based on the importance of the node in the network and the nodes it is connected to. It can be expressed as:

$$\begin{array}{c} PR_{i}=\left(1-d\right)+d*\sum_{j\in M_{i}}\frac{PR_{j}}{k_{j}} \end{array}$$ (8)

where PR_*i*_ is the PageRank of node i, d is the damping factor (usually set to 0.85 as it helps to balance the importance of in-links and the random jumping behavior), M_*i*_ is the set of nodes with an edge pointing to node i, PR_*j*_ is the PageRank of node j, and k_*j*_ is the out-degree of node j. To draw the comparison between agricultural and non-agricultural trade, we compute the time-averaged centrality measures for each state. We also evaluate the network centrality measures, including betweenness and degree centrality, to identify the existence of hubs.

Finally, we explore the statistical relationship between mean in-degree (X) and out-degree (y), where mean in-degree and mean out-degree are calculated over the study period for each state. We extend this analysis to understand the statistical relationship between trade time-averaged import/export value and their trend with population and GSDP at PPP’s value and trend, respectively (a detailed explanation is given in the S1 File)

## Results

In the DITN analysis, we classified the network into agriculture and non-agriculture classes based on the types of commodities. Fig 1 shows the overview of interstate trade in India. We first consider the overall trading connection of agricultural and non-agricultural commodities with average over the temporal window. We show the value of commodities traded between each state and the total import and export commodities traded through each state. We use a similar approach to perform analysis for physical values (weights) of commodities and also for inflation-adjusted monetary values. A detailed explanation of the calculation and results are shown in the S1 File

### Agricultural trade

Fig 1a depict the average transfer of agricultural products across the Indian states for the period 2010–2018. Each year, the aggregated volume of food flow—in monetary value—is around ₹65.5 billion. The average exports and imports of agriculture commodities of various states are presented in Fig 1c and 1e, respectively. Further, Table 2 represents the top five exporters and importers in the agriculture DITN, along with the corresponding mean trade value (in ₹). The North Indian states: Punjab (PN), and Haryana (HR), along with Madhya Pradesh (MP), Andhra Pradesh (AP), and Chhattisgarh (CH), are the key food suppliers in India, contributing with more than 75% of agricultural products export within India. Whereas the states like Tamil Nadu (TN), Maharashtra (MH), Uttar Pradesh (UP), West Bengal (WB), and Bihar (BR) import 50% of the worth of annual agricultural products.

**Table 2:** Top five states with highest exports and imports for overall agriculture trade and non-agriculture trade. The full name of the states is mentioned in the abbreviation section.

S_(*in*)_	Weight (₹billion)	S_(*out*)_	Weight (₹billion)
Agriculture trade			
TN	8.49	PN	20.82
MH	7.15	HR	9.34
WB	6.76	MP	8.96
UP	6.25	AP	5.58
BR	5.74	CH	4.80
Non-Agriculture trade			
MH	55.55	OD	96.45
WB	53.25	CH	71.87
AP	43.73	AP	60.92
UP	39.10	JH	48.01
KA	35.85	KA	34.30

### Non-agricultural trade

The non-agricultural commodities mainly include infrastructure-supportive commodities like coal, steel, cement, mineral ores, and fertilizers. The total trade value of non-agriculture commodities in DITN is around ₹462.48 billion, significantly larger than the agriculture products’ trade. Fig 1b demonstrates the transfer of major non-agriculture products across the Indian states for 2010–2018. (Fig 1d and 1f) shows the mean exports and imports of non-agriculture goods. Chhattisgarh (CH) and Odisha (OD) contribute 35% of exports of total non-agriculture trade in DITN. At the same time, WB and MH are leading importers (Table 2).

Summarizing above, overall, the northern Indian states Punjab (PN) and Haryana (HR) dominate the agriculture DITN, while the south Indian states Tamil Nadu (TN) to Andhra Pradesh (AP) is the most prominent link for trade transfer with the average trade value of 3 billion ₹yr^−1^, which accounts for 4.5% of the total trade volume/value of the network (Fig 1a). In contrast to the agriculture trade, the non-agriculture trade does not exhibit a dominant link (Fig 1a and 1b). The states considered for this analysis significantly participate in transferring non-agricultural commodities, albeit unevenly. The flow of non-agricultural goods from OD to WB is the link with high trade transfer with the mean annual trade value of 20.75 billion ₹yr^−1^ which comprises 4.5% of total non-agriculture interstate trade (Fig 1b).

### Temporal changes in the topological characteristic of interstate trade network

**Fig 2:**
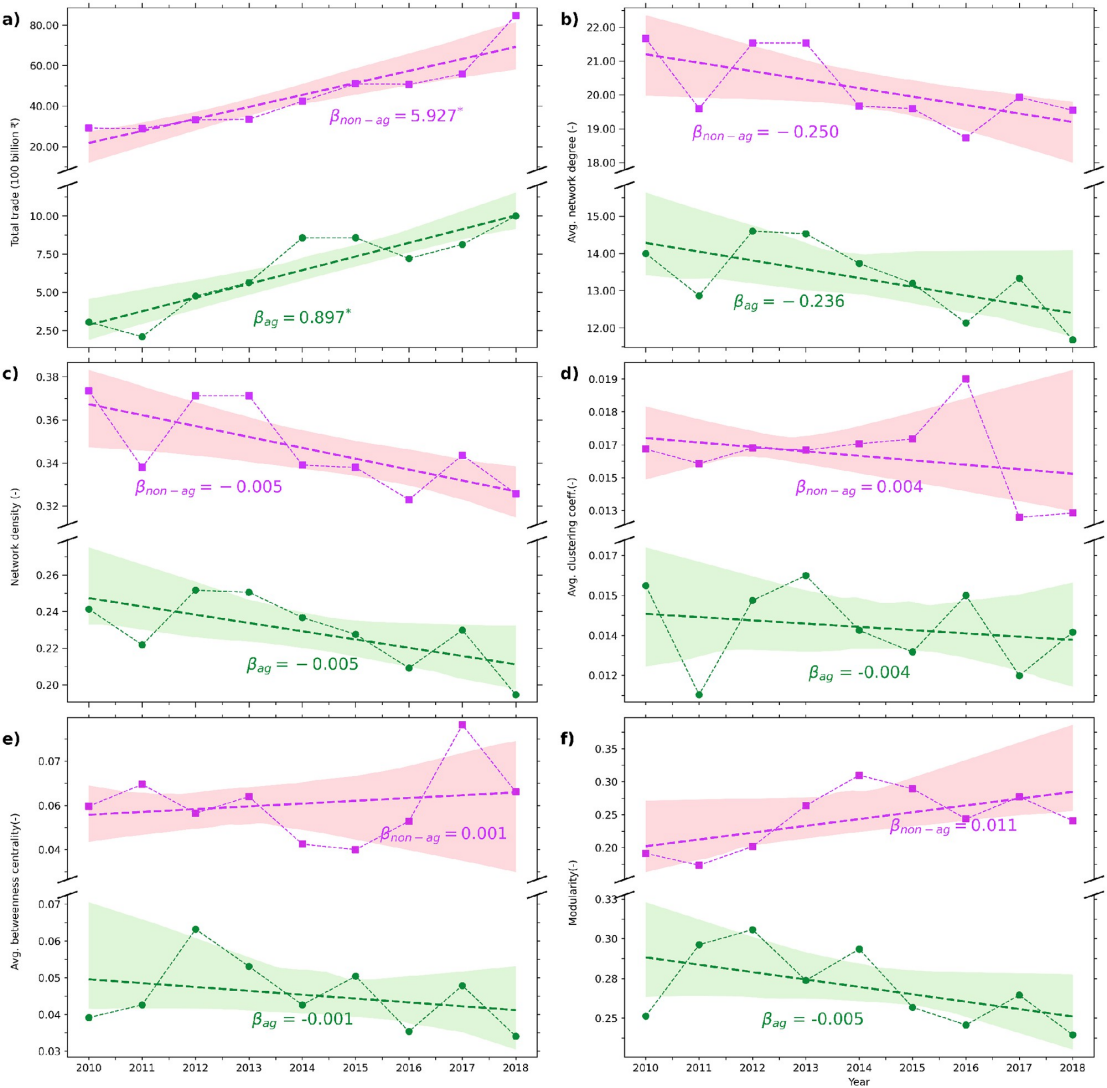
Temporal changes in total trade value and topological characteristics of the network. (a)Total traded value (in ₹100 billion), (b) Average network degree, (c) Network density, (d) Average clustering coefficient, (e) Average betweenness centrality, and (f) modularity for 2010–2018. here, *β* indicates the slope of a linear trend line fitted to scattered points (values with * represent a significant trend at *p* − *value* < 0.05). The results are presented for the Agriculture (green) and Non-Agriculture (purple) interstate trade networks.

To understand the networks’ structural changes over the temporal window of 2010–2018, we use the topological parameters of a complex network as detailed above (See Method section). Over the last decade, the number of nodes participating in agriculture (and non-agriculture) trade increased from 26 (28) in 2010 to 27 (30) in 2018, respectively. In the case of links, non-agricultural trade links reduced from 325 to 303, whereas the links in the agricultural trade decreased from 210 to 181 in the time frame of 2010–2018 (S2 Table in S1 File). Fig 2a describes India’s temporal change of agriculture and non-agriculture spatially-averaged interstate trade value. The traded values of agriculture and non-agriculture trade increased rapidly, exhibiting a significant (*p* − *value* < 0.05; linear trend) change with time. The total traded average annual values increased by 23.3% and 15.4% for agriculture and non-agriculture trade, respectively. The strong trend (*β*_*non*−*ag*_ = 5.93 billion ₹yr^−1^) is observed for the non-agriculture trade, whereas agricultural trade has gradually increased with a dip (16%) in recent years. This decrease may mark the impact of 2016–2018 droughts in India [58]. It is noted that these conclusions are drawn using finite data points owing to limited observations available. The network’s topological properties do not significantly change over the study period (Fig 2b). However, its relation to trade value is critical to analyze. Fig 2b elaborates on the change of average degree over time for spatially-averaged agriculture and non-agriculture trade. The average degree of non-agriculture trade observes a modest decrease (*β*_*non*−*ag*_ = −0.25), with an increase in trade flow. In the agriculture trade, the slight decrease (*β*_*non*−*ag*_ = −0.24) in the average degree also follows the same pattern. Note that in our analysis, we considered trades only via railways which is the preferred mode of transport of bulk substances, although the neighboring states may prefer to choose other transportation modes (via roads). The network density (Fig 2c) displays the weak negative trend in spatially-averaged agriculture trade (*β*_*non*−*ag*_ = −0.005), and in the case of non-agriculture trade, the pattern is the same. Increasing total trade with no significant change in network degree depicts the diversification of commodities over the network’s existing connections.

Along with the increasing trend in spatially-averaged non-agricultural commodities, the positive trend of the clustering coefficient shows that the connectedness of export lines has improved over time (Fig 2d). Average betweenness centrality shows little positive change in non-agriculture, whereas a modest decreasing trend in spatially-averaged agriculture trade (Fig 2e). This indicates that for non-agriculture trade, fewer paths are available for a commodity to transfer, leading to a more centralized system that relies only on a few key states, whereas the agriculture trade network has more links to transfer the goods, leading to more efficient and less congested network system. In the case of modularity, the spatially-averaged agricultural network shows a low decreasing trend while non-agriculture depicts a modest, increasing trend (Fig 2f). Increasing trade with increasing modularity in spatially-averaged non-agriculture trade over time indicates the new connection in existing communities and the formation of new communities with less connectivity, reflecting that the network is becoming more fragmented and that the flow of trade resources within the network is fragile to disruption. On the other hand, a contrasting result is observed in the trade network of spatially-averaged agriculture commodities, where decreasing modularity with increasing trade signifies the dissolution of communities and the formation of a more homogeneous network.

### Trade and network properties’ variation with space and time

**Fig 3:**
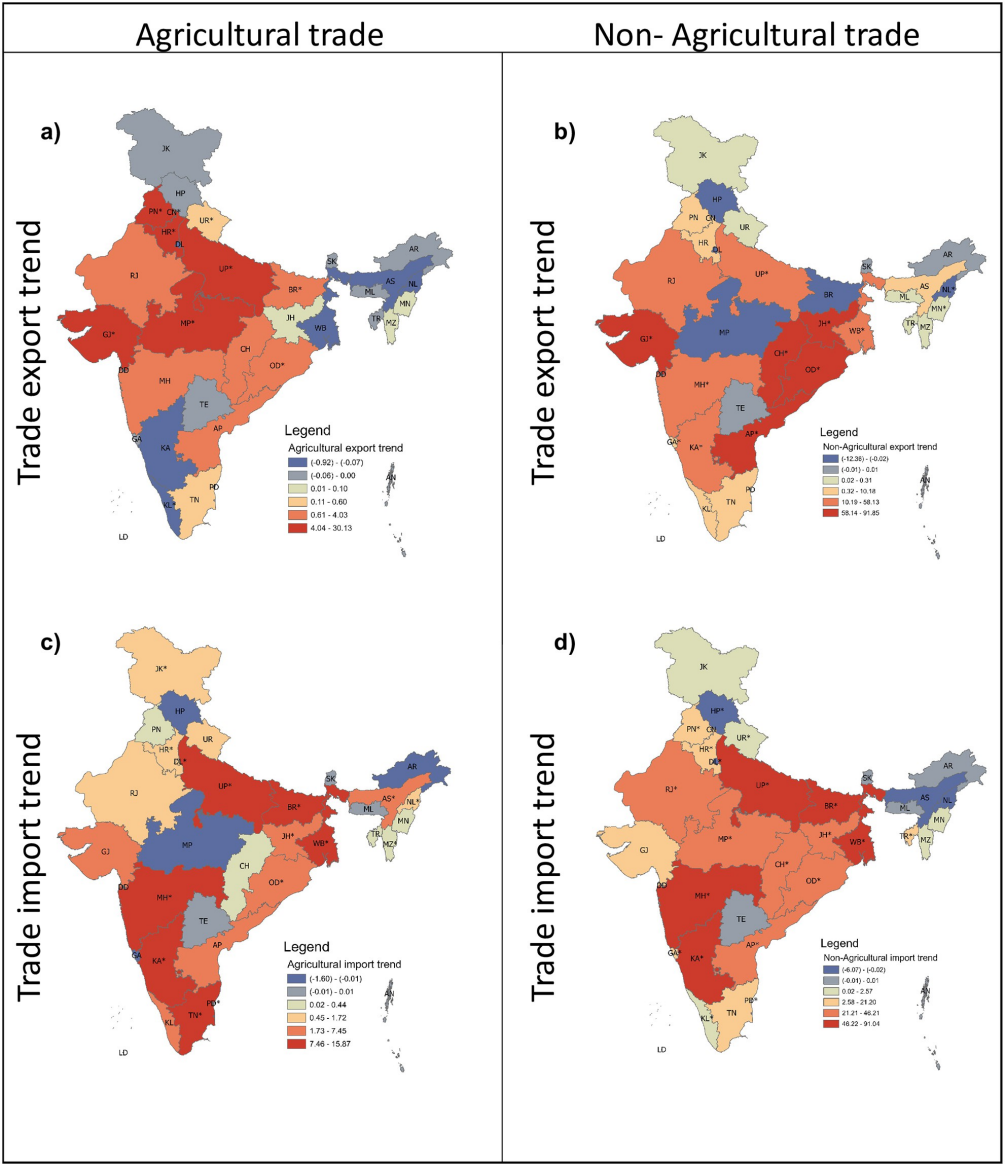
Spatial variation in the trends of exports and imports. (a and c) shows the spatial variations of export and import for agricultural products through trade trends, whereas (b) and (d) present the same for Non-agriculture commodities. Here the states with * represent states with a significant trend (at p-value < 0.05) of commodities over the period of 2010–2018. All values are in billion ₹.

We analyze the spatial variation of the trends for import and export in agricultural and the non-agricultural trade network. Fig 3a emulates the spatial variation in export trends of agricultural commodities. The major exporters of agricultural products, such as PN, HR, UP, MP, and GJ, have significantly increased their exports. The states, namely Karnataka (KA), Nagaland (NL), Kerala (KL), and Assam (AS), displayed a strong negative trend in exporting agricultural products. In the case of the import of agricultural commodities, MP is the only state that has reflected a negative import trend with increasing export (Fig 3c). The number of states having a negative trend in the export of non-agriculture commodities is high compared to agriculture commodities (Fig 3b). On the other hand, contrasting result is observed in the import of non-agriculture commodities as the number of states with positive trends in non-agriculture import is more than that of the import of agriculture commodities (Fig 3d).

**Fig 4:**
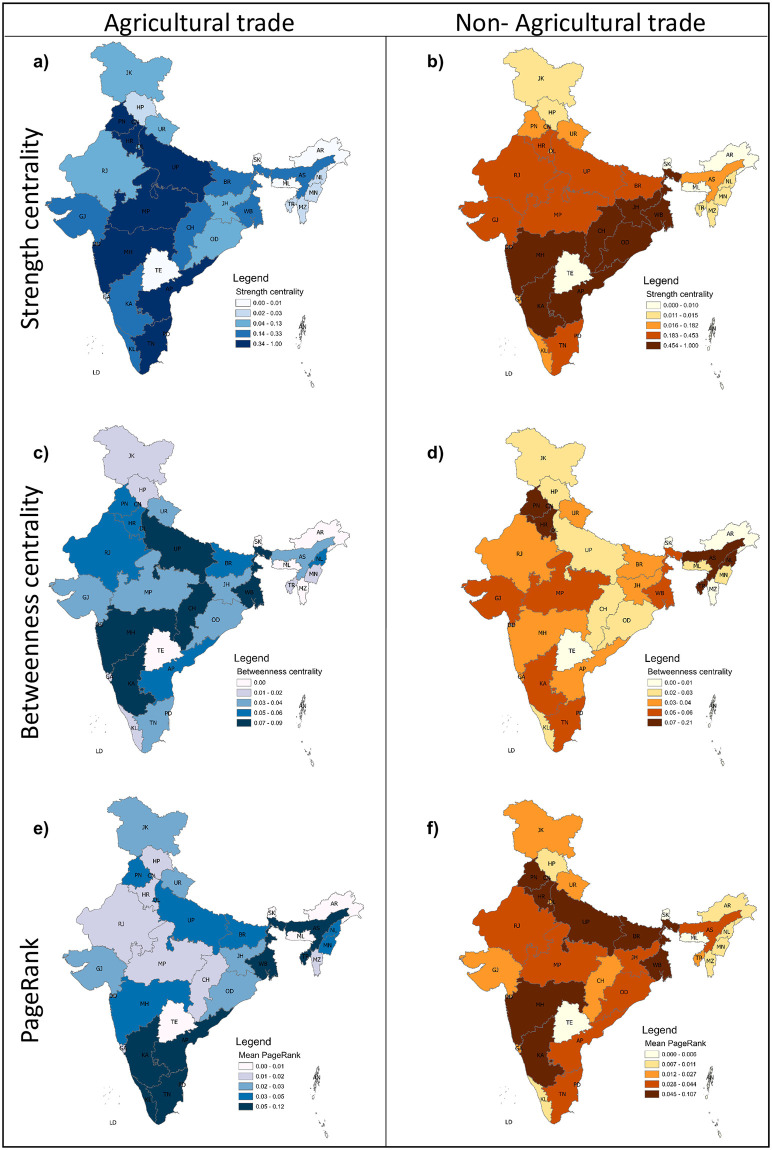
Relative influence of states on interstate trade network. (a) and (b) shows the time-averaged strength centrality of each state for agriculture and non-agriculture DITN, respectively, (c) and (d) depict the time-averaged betweenness centrality for the same. In comparison, (e) and (f) indicate the time-averaged PageRank centrality of each state.

The centrality measures effectively recognize the state’s relative influence in the network. Here we use time-averaged strength, betweenness, and PageRank centrality to capture the highly influential states in the interstate trade network for agriculture and non-agriculture commodities (see the Method section for details). The time-averaged strength centrality combines the in-strength and out-strength of the individual state in the network (Fig 4a and 4b). The northern states PN, HR, UP, and the southern state AP are influential in agriculture trade based on their high trade volume and connections of trading (number of connections per state). On the other side, in the case of non-agricultural commodities, OD, CH, JH, and AP have higher trade values and connections. Though the volume of trade with their connection matters in finding the hubs in the system, it is also essential to look into the structure of the network and how they are connected.

Fig 4c and 4d show the time-averaged betweenness centrality of each state for agriculture and non-agriculture DITN. The high betweenness centrality in UP, MH, CH, and WB in agriculture DITN; and PN, HR, AS, and DL in non-agriculture DITN suggest that they are connected to many states through the shortest path with respect to their trading link. The states with high betweenness act as major connection nodes that control the trade flow between different parts of the network, with the majority of trade passing through them with the shortest path connection. The time-averaged PageRank centrality of agriculture and non-agriculture DITN (Fig 4e and 4f) show that states like TN, AS, KA, and KL in agriculture and WB, UP, PN, and BR are well-connected to other important states in the network that have high connectivity.

Here, we also explore the correlation between betweenness centrality and weighted degree centrality to identify the hubs and importance of states in the trade network. (Fig 7a in S1 File) shows a correlation between degree centrality and betweenness centrality in agricultural trade transfer for the entire study period. We note that for agricultural commodities, states with a high degree centrality (number of connections to other states) also exhibit high betweenness centrality, which indicates that they have many direct connections to other states and also appear on many of the shortest trading connections between other states, making them important for the overall flow of commodities in the network. However, non-agricultural trade shows no obvious correlation between node betweenness and degree centrality (Fig 7b in S1 File).

**Fig 5:**
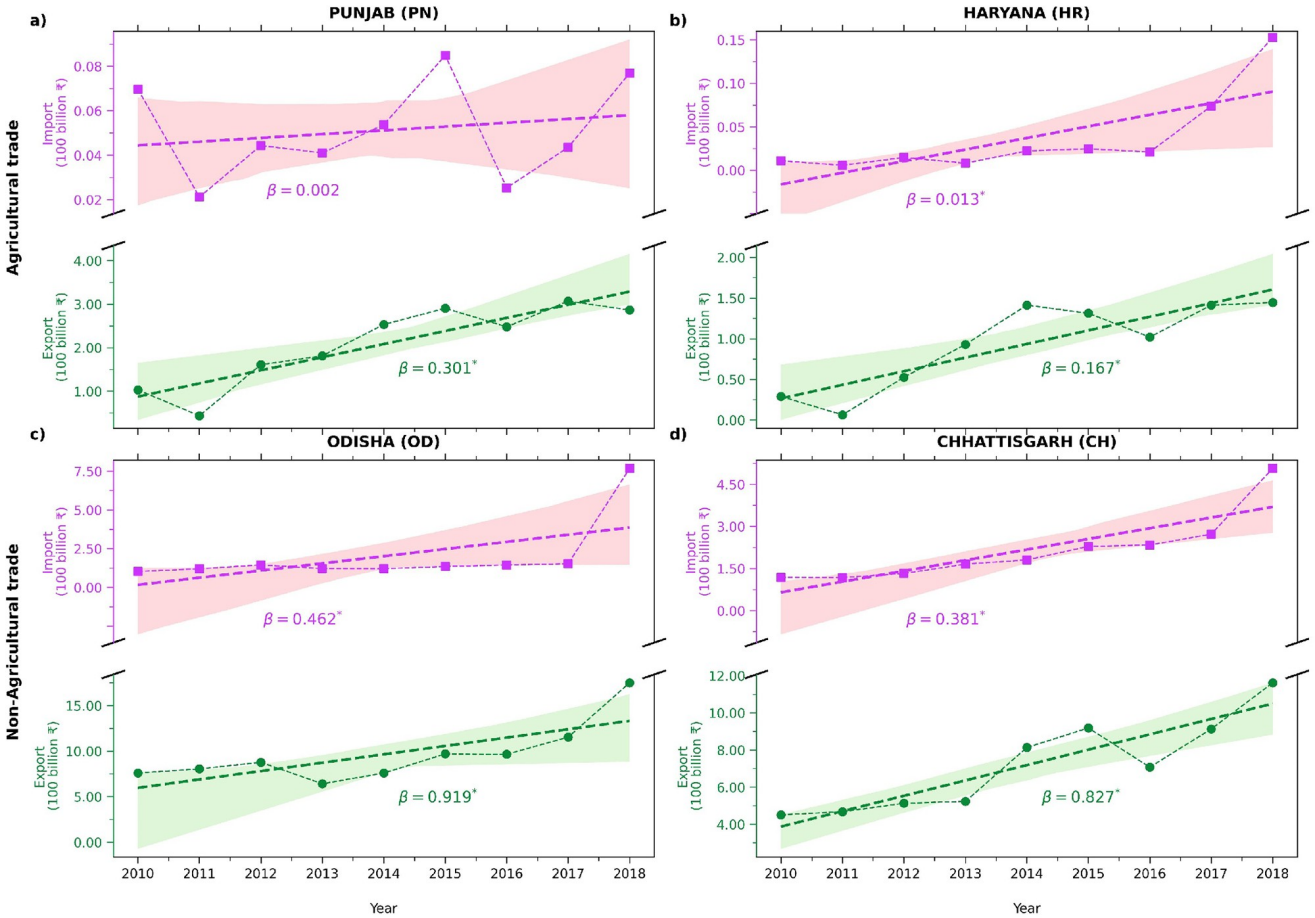
The temporal changes in the trade value of leading exporters. The temporal variations of exports and imports of leading exporters of agriculture (Punjab and Haryana) and non-agriculture (Odisha and Chhattisgarh) commodities over the period 2010–2018. Also shown are the respective linear regression slope (*β*).

We then evaluate the temporal variation of the top two major exporters of agriculture and non-agriculture trade concerning their respective imports and exports. Fig 5 presents the temporal change of (PN and HR) that have contributed significantly to exporting agricultural commodities. Both PN and HR have steadily increased import and export trade as trades have not changed significantly over time, making them more stable. While leading exporters in non-agriculture commodities, OD and CH have increased imports and exports. The result can be attributed to the states being pioneers in processing metallurgical-dominated products, which require the high import of raw products and can export the finished products [59] (Fig 5c and 5d).

**Fig 6:**
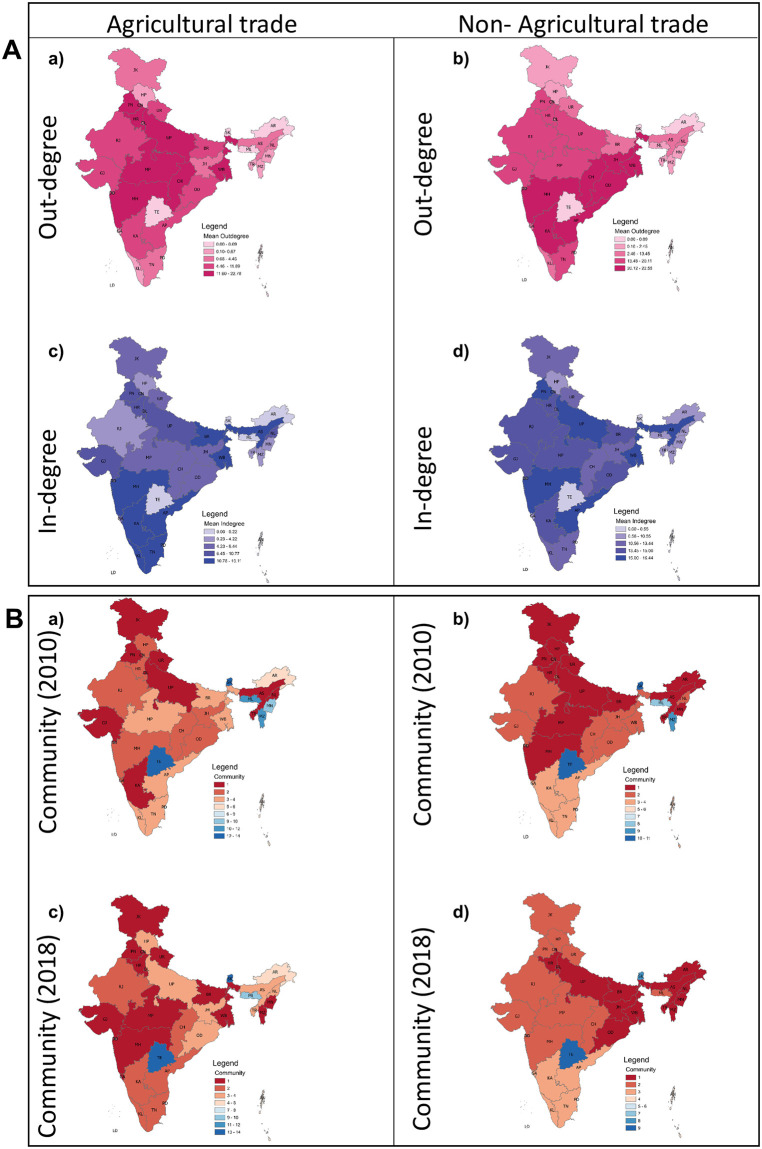
Spatial variations of in-degree, out-degree and community. Study area showing average out-degree and average in-degree, respectively, for agriculture (A—(a and c)) and non-agriculture (A—(b and d)) commodities. All averages are calculated from 2010 to 2018. (B—(a and c)) show the community formation of agriculture, while (B—(b and d)) depict the communities in non-agriculture trade for 2010 and 2018, respectively.

Finally, we use the time-averaged in-degree and out-degree parameters’ and analyse their spatial variation for both agricultural and non-agricultural commodities to understand the variation in import and export trade connections. Fig 6A(a) and 6A(c) depicts the number of outgoing and incoming trade flows over the analysed time period. The north-central belt of India has shown prominent participation in the export trade, whereas the southeastern part of India has a higher connection in importing agricultural commodities. This is due to the major exporter state of agriculture trade being situated in the north-central belt, which has high production of agricultural commodities. The non-agriculture trade network, on the other side, has shown the clustering pattern in the export connections (Fig 6A-(b)) that suggests their states’ geographic location influences the non-agriculture trading. For example, Fig 6A-(b) shows that the states in the central and east central belt of India (including OD, CH MP, and MH) have a higher connection in commodity export, whereas there is a spatially distributed pattern of states with high commodity import connections (Fig 6A-d).

We also evaluate the spatial changes in the formation of communities (states with densely connected trade links) over time. Fig 6B-(a) and 6B-(c) represents the community change in the agriculture trade network from the year 2010 to 2018. Here (Fig 6B-(a)) shows that in the largest community, the JK, PN, UR, UP, GJ, KA, NL, and AS states are well connected internally through trading in 2010, then other states. Fig 6B-(b) and 6B-(d) show the community change of non-agricultural commodities’ trade. The change in community depicts the change in the trading connection between different regions of the trade network. The community number in (Fig 6B) provides information about states in the community that have dense connections among them compared to other states. We observe that non-agriculture communities are changing from the north-central belt to the east-central belt, reflecting that states changed their trade connectedness towards major exporters of non-agriculture commodities, including OD and CH.

### Statistical relationship between import and export commodities in DITN

**Fig 7:**
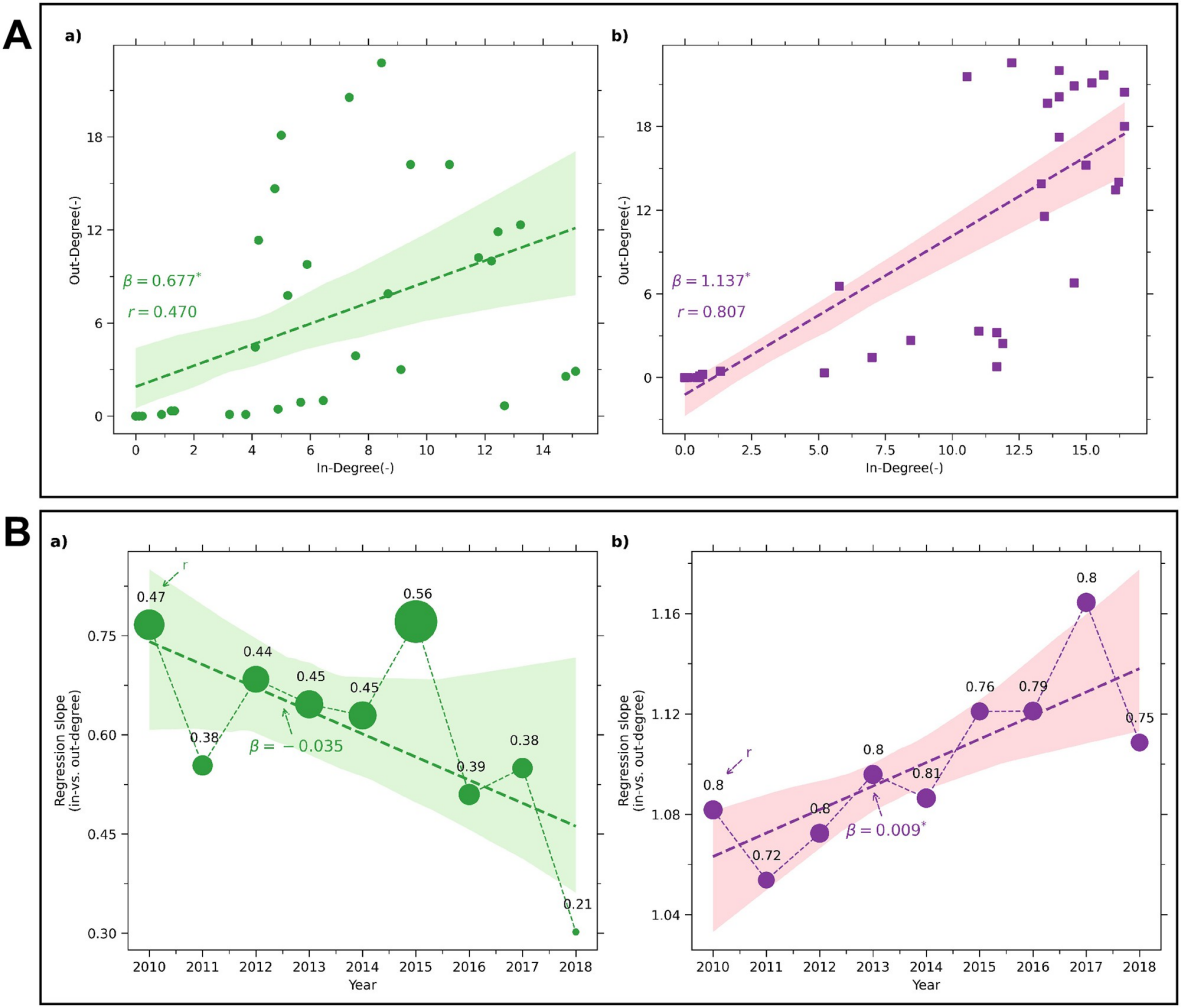
Spatio-temporal statistical relationship between import and export commodities. (A) Scatter plots of time-averaged in-degree and out-degree over nine years (2010–2018) for Indian states. *β* indicates the slope of the fitted linear regression line to scattered points, and *r* indicates the Pearson correlation coefficient. (B) Scatter plots of the year-wise slope of in-degree and out-degree. The size and numerical above the marker represent the respective correlation value. The fitted regression line (dash line) and corresponding 95% confidence band are shown in all panels. The results in sub-panels (a) and (b) are for agriculture trade and non-agriculture trade, respectively.

We analyse the statistical relation between in-degree and out-degree. Fig 7A shows the relationship between the import and exports of agriculture and non-agriculture commodities for each state, based on the time-averaged in-degree and out-degree parameters. The trade distribution by states in agriculture products with respect to their incoming and outgoing trade connections is sparse (*β* = 0.67) and has a moderate correlation (*r* = 0.47). This indicates that no major hotspots of states dominantly govern the trade flow in the agriculture trade network (Fig 7A-a). However, in the non-agriculture trade, the states with high trade export connections also have high trade import connections (*β* = 1.14 and *r* = 0.81), making them strongly connected in the trade network and hotspots for transferring commodities (Fig 7A-b).

Fig 7B indicates the annual temporal change of a network for its import and export commodity transfers, represented here as the corresponding regression slope between the in-degree and out-degree of the network. The agricultural interstate trade network shows a negative trend (*β* = −0.035) in trade connections and has low correlation values over time. This indicates that the states are not dependent on hotspot regions, reflecting that the states are becoming more self-reliant over the years through diversification, possibly to reduce the future food risk (Fig 7B-a). In contrast, the non-agricultural interstate trade network has maintained a slightly positive trend (*β* = 0.009)in making more trade connections over the years. In general, with an overall high correlation value throughout the temporal window, the non-agricultural trade network appears to be highly connected and dependent on the hot spots making them vulnerable to external disruption, which is also observed in their trend analysis of topological characteristics (Fig 7B-b).

We evaluate the statistical relationship between import/export trade values and population/GSDP at PPP. We observe no significant correlation between the time-averaged export and population for agricultural and non-agricultural trade networks (Fig 12: a,b,e and f in S1 File). In contradiction, there is a high correlation between the time-averaged trade import with population and GSDP at PPP, respectively (Fig 12: c,d,g and h in S1 File). In addition to that, we also quantify the correlation between the import/export commodity trend and population/GSDP at PPP trend (Fig 13 in S1 File). The population trend shows a high and positive correlation with trade export in the leading trade exporter states, including Punjab and Haryana for agricultural trade and Chhattisgarh, Jharkhand and Odisha for non-agricultural trade. The result indicates that population growth leads to an increase in resource demand, which can drive the growth of export-oriented industries (Fig 13: a and b in S1 File). However, it is important to note that correlation does not necessarily imply causation. Other factors may contribute to both population growth and trade export growth, such as favourable trade policies, technological advancements, or the availability of natural resources, which also should be taken into account. We also observe the clustering of states which exhibit a high and positive correlation in the trend of import commodities trade with population and GSDP for non-agricultural DITN (Fig 13: d and h in S1 File). This is because only specific states have resources for non-agricultural commodities and refineries for manufacturing the products, and other states have to import these commodities to satisfy their demand.

## Discussion

The advancement in transport infrastructure and liberal trading policies have enhanced the spatio-temporal import and export at an international scale [60, 61]. The community has access to the resources they cannot produce and can procure the commodity through trade, leading to over-dependence on global trade. However, rising economic instability, disasters, and geopolitical situation infuse the fragility in the international trade network [16, 62]. Thus, maintaining a robust domestic interstate trade network is essential with global interconnectivity to reduce the impacts of such external shocks. This study evaluates the spatio-temporal characteristics of agriculture and non-agriculture (domestic) interstate trade networks across India over the period 2010 to 2018 using a complex network approach. The key contributions of the study going beyond the previous studies [2, 19, 22, 63–67] by exploring the underlying characteristics and nuances of DITN. The spatio-temporal change of the agriculture versus non-agriculture trade provides crucial insights into changing nature of the commodities based on their class for authorities and policymakers to manage interstate trading.

While International trade networks and single commodity global trade networks have received significant attention [1, 26, 29, 57, 68, 69], the single commodity interstate trade network is rarely focused [70–73]. Understanding the evolution of interstate trade networks of multiple commodities can help us gain insights into the nation’s self-sufficiency and identification of key hubs that are the most critical to sustaining the supply chain [21, 74, 75]. This study provides a detailed analysis of the India-wide pattern and spatio-temporal variation of the diverse resources over the last decade. It reveals that non-agriculture and agriculture trade show contrasting results concerning their topological characteristics and trade values. In the case of non-agriculture DITN, no significant network density change with an increase in clustering coefficient signifies the improvement in connectedness over time, and the major trading states are relatively close in the existing trading route. With this, there is also an increase in modularity, which suggest that the network is becoming more fragmented. The fragmentation of non-agriculture DITN suggests that the trading is divided into several groups of trading states and is less connected to each other, which decreases their resilience to shocks [76, 77]. In contrast, agriculture DITN, with a modest decrease in network density, modularity, and clustering coefficient with increased trade, depicts that trade is less fragile to shock, which is also reflected through decreasing average betweenness centrality as it suggests that trade is becoming more direct and less reliant on hubs. Indicating that agriculture DITN is less prone to external shock and the network can recover quickly [76].

We also observe the same characteristics while examining the trade’s import and export connectivity in both networks. While non-agriculture DITN is moving towards becoming more dependent on hubs, agriculture DITN shows contradictory characteristics by diversifying the trade connection and becoming less dependent on hubs. This result is attributed to the specific state having resources for non-agriculture commodities and refineries for manufacturing those commodities. The finding can be attributed to the quick recovery of trade due to the self-reliant agriculture trade network during external shocks where interstate movements were minimal.

Our analysis enables us to quantify the contribution of nodes and the temporal change of trade connection over a temporal window in agriculture and non-agriculture DITN. We note that importing non-agriculture commodities has more positive spatial growth than agriculture commodities due to economic growth in the sector and that enhanced the strength between the existing linkages of trade [78–80]. This implies more network dependence on non-agriculture exporting states over a temporal period. Whereas in agriculture trade growth, export is limited to the northern belt of India. Diversification in agricultural trade can be achieved through alternative crop production or decentralizing the production of various crops [81, 82]. In the case of non-agricultural trade, decentralization is challenging due to the natural constraint imposed by the availability of natural resources within the Nation.

In addition to that, time-averaged agricultural and non-agricultural import trade for different states exhibits a high correlation with population data and with the GSDP at PPP. This gives an insight that states with higher population and purchasing power tend to import more commodities than those with low income and population. In addition, we also observe the positive correlation between the export and population for leading exporter states in the agricultural and non-agricultural trade network, which may be attributed to increased production in exporting states and demand in importing states. We note that translating the correlations to causality requires further analysis, which is beyond the scope of this study.

In order to obtain a more comprehensive picture of national trade networks, future works need to address the following limitations. (a) We consider the commodities transfer through railways. Though a significant portion of the transfer of commodities is traded through railways in India [61], considering commodities through air, water, and road transport will make the analysis complete and more robust. (b) Here we consider aggregated data on agriculture and non-agriculture commodities. However, studying the individual commodity transfer (i.e., rice, wheat, coal, and metal products) and their evolution can bring new insights. (c) The number of data points considered for trend analysis is limited in this case owing to the limited data available from sources. Hence, future studies need to consider longer duration time series for robust estimation of parameters of interest. (d) For the agriculture trade network, as India is self-reliant on agriculture production and we also observe that major trading hubs are also major production hubs, it is safe to assume that the redistribution of resources originates from India only. Whereas in the case of non-agricultural networks, due to data unavailability, redistribution of resources from outside India is not considered.

Trade globalization has shown an increase in food resilience and water security, whereas it also enhanced the likelihood of global crises due to growing dependence on major production hubs [74, 83, 84]. Overall, the topological quantification of Indian inland trade through a complex system’s lens could help to understand the trade network’s resilience and recovery by identifying the network’s important and vulnerable sections to external disruptions. Our study of interstate trade at a local scale can be extended to understand virtual water trade. The virtual water traded through agriculture and non-agriculture commodities with the topological properties of the network and their evolution can help understand impacts on the local water system and aid in making informed decisions on the issue of water and food security [64, 74, 85–87].

## Conclusions

We studied the spatiotemporal changes in agricultural and non-agricultural interstate trade networks comprising several commodities, which is vital to understand commodity transfer at a national scale. Some key messages deserve attention when looking into the interstate trade network of India.

- The result shows that agricultural and non-agricultural trade networks have distinct characteristics. The agricultural trade is moving toward self-reliance, as indicated by the decreased network density, modularity, and clustering coefficient despite the increase in trade volume. In the case of non-agricultural trade, the network is becoming more fragmented and, therefore, may be prone to external shocks.
- The non-agriculture trade is moving towards over-dependency on the leading exporter states in trade. This is due to the specific states having resources for non-agricultural commodities and refineries for manufacturing the concerned products, which indicates that any external shock or disruption in trade relations with these states can have severe consequences on domestic non-agricultural trade flow. On the contrary, the agriculture trade is less dependent on leading exporter states through diversifying the trade flows. This indicates that during external shocks, quick recovery of trade flow is possible due to self-reliant agricultural trade.
- We observe that the GSDP at PPP and population play a major role in the import trading pattern of individual states for agricultural and non-agricultural interstate trade transfer. This suggests that the states with higher purchasing power and population dominate the import of agriculture and non-agriculture commodities trade.

These points, in addition to suggesting new insights, also act as the first step in understanding the Indian interstate trade system. which opens new avenues and questions for future research. The indication of quantity-based commodities with their monetary values gives us the first insight into the inter-India trade pattern among different states. It can be extended to understand food transfer, nutrient pollution transfer, virtual water transfer, and so forth through trading that can help understand the nation’s food and water security.

## Supporting information

S1 File This file contains additional text, figures, and tables to interpret results and discussions. (PDF)

## Abbreviations

AP: Andhra Pradesh
AR: Arunachal Pradesh
AS: Assam
BC: Betweenness Centrality
BR: Bihar
CH: Chhattisgarh
CN: Chandigarh
DD: Daman and Diu
DGCI&S: Directorate General of Commercial Intelligence Statistics
DITN: Domestic Interstate Trade Network
DL: Dehli
GA: Goa
GJ: Gujarat
HP: Himachal Pradesh
HR: Haryana
JH: Jharkhand
JK: Jammu and Kashmir
KA: Karnataka
KL: Kerala
MH: Maharashtra
ML: Meghalaya
MN: Manipur
MP: Madhya Pradesh
MZ: Mizoram
NL: Nagaland
OD: Odisha
PN: Punjab
RJ: Rajasthan
SK: Sikkim
TN: Tamilnadu
TR: Tripura
TS: Telangana
UP: Uttar Pradesh
UR: Uttarakhand
WB: West Bengal
