# Supplementary material for: Tracing spatiotemporal changes in agricultural and non-agricultural trade networks of India
Source: PLoS One. 2023 Sep 26;18(9):e0286725. doi: 10.1371/journal.pone.0286725 (PMC10521996; doi:10.1371/journal.pone.0286725)
Supplement: S1 File — (PDF) [file pone.0286725.s001.pdf]

Supplementary information for *Tracing Spatiotemporal Changes in Agricultural and Non-agricultural Trade Networks of India*

Sujata Kulkarni[1◑], Raviraj Dave[1◑], Udit Bhatia[1,2*] Rohini Kumar[3*],

1 Discipline of Civil Engineering, Indian Institute of Technology Gandhinagar, Palaj, Gandhinagar, Gujarat -382355, India
2 Dr. Kiran C. Patel Center of Sustainable Development, Indian Institute of Technology Gandhinagar, Palaj, Gandhinagar, Gujarat -382355, India
3 Computational Hydrosystems, Helmholtz Centre for Environmental Research, UFZ, Leipzig, Germany

◑These authors contributed equally to this work.
* Corresponding authors: bhatia.u@iitgn.ac.in; rohini.kumar@ufz.de

1 Trade network based on the physical values (weights) of commodities

In this study, we also analyzed the trade flows based on their physical values (weight) [1]. The trade transfer between two states is represented in quintals. In this analysis, we followed the same procedure as in the manuscript without considering the monetary value of each commodity. The Fig 1 to Fig 6 depicts the analysis for physical value.

2 The monetary value of commodities

The monetary value of each commodity is provided in Table 1. Here, we consider the minimum support price (MSP) for agricultural commodities. Whereas in the case of non-agricultural commodities, the retail price is considered.

3 Temporal changes in network properties and metric network centralization

The temporal changes in the network properties for agricultural and non-agricultural trade are provided in Table 2. The table shows the changes in the nodes and links for the temporal window of 2010 to 2018.

We use network centralization metrics to understand the existence and prevalence of hubs in the agricultural and non-agricultural DITN. We calculate the weighted degree centrality and betweenness centrality for each state from 2010 to 2018 and plot them to understand the relationship and the existence of hubs Fig 7.

4 Trade network with inflation-adjusted monetary values

We also analyze the monetary value of commodities with an inflation adjustment to provide the actual growth of the trade network in agricultural and non-agricultural

commodity transfer. We use the consumer price index as an indicator for calculating the adjusted value of the corresponding year. The mathematical representation is given in (Equation 1).

$$AdjustedValue_j = \frac{Value_i \times CPI_i}{CPI_j} \tag{1}$$

Where j represents the current year, and i depicts the original year, CPI is the consumer price index for different commodities. We obtain the CPI data for the temporal window of 2010 to 2018 from the Reserve Bank of India (RBI) [2].

We replicate a similar analysis of trade transfer for inflation-adjusted monetary value. The Fig 8 to Fig 11 shows the result for inflation-adjusted trade transfer.

5 Statistical relationship between trade transfer, Population and Gross state domestic product

We explore the statistical relationship of the Domestic Interstate Trade Network (DITN) with non-topological parameters, including states' population and Gross State Domestic Product (GSDP). The latest demographic data is available from the 2011 census, which may not precisely depict the present population and the population's variation in the study period. To address this issue, We obtain the population data of each state for the time frame of 2010 to 2018 from the Worldpop data repository at a spatial resolution of 1 km [3]. We then aggregate the population data for each state through zonal statistics in ArcGIS. We analyse the statistical relationship between time-averaged population with time-averaged trade import and export for each state in agricultural and non-agricultural trade networks Fig 12. The statistical relationship of import and export trend with population trend is also analysed for individual states and shown in Fig 13.

In the case of GSDP, we collect the individual state's GSDP from RBI [2] for the period of 2010 to 2018. To compare the actual growth of the GSDP, we convert the GSDP value to GSDP at Purchasing Power Parity (PPP), which indicates the purchasing power of an individual state. To calculate the GSDP at PPP, we use the annual exchange rate of INR (₹) to USD ($) and the PPP conversion factor obtained from the world bank [4]. The calculation of GSDP at PPP is shown in (Equation 2).

$$GSDP@PPP_{y,s} = \frac{GSDP_{y,s} \times P_y}{E_y} \tag{2}$$

Where y depicts the year, s represents the state, P is the PPP conversion factor, and E is the exchange rate.

We follow a similar approach to identify the statistical relationship for the time-averaged value of GSDP at PPP against the trade import and export for both agriculture and non-agriculture trade as we did in the case of the population (Fig 12). We also quantify the statistical relationship of the import and export trends with GSDP at PPP trend (Fig 13).

References

1. Ministry of Commerce and Industry. Directorate General of Commercial Intelligence and Statistics; 2021. `http://www.dgciskol.gov.in/`.

2. Reserve Bank of India. CPI - Rural, Urban, Combined (All India); 2023. https://dbie.rbi.org.in/BOE/OpenDocument/1608101727/OpenDocument/opendoc/openDocument.faces?logonSuccessful=true&shareId=1.

3. Tatem AJ. WorldPop, open data for spatial demography. Scientific data. 2017;4(1):1–4.

4. The world bank. PPP conversion factor, GDP(LCU per international $; 2023. https://data.worldbank.org/indicator/PA.NUS.PPP.

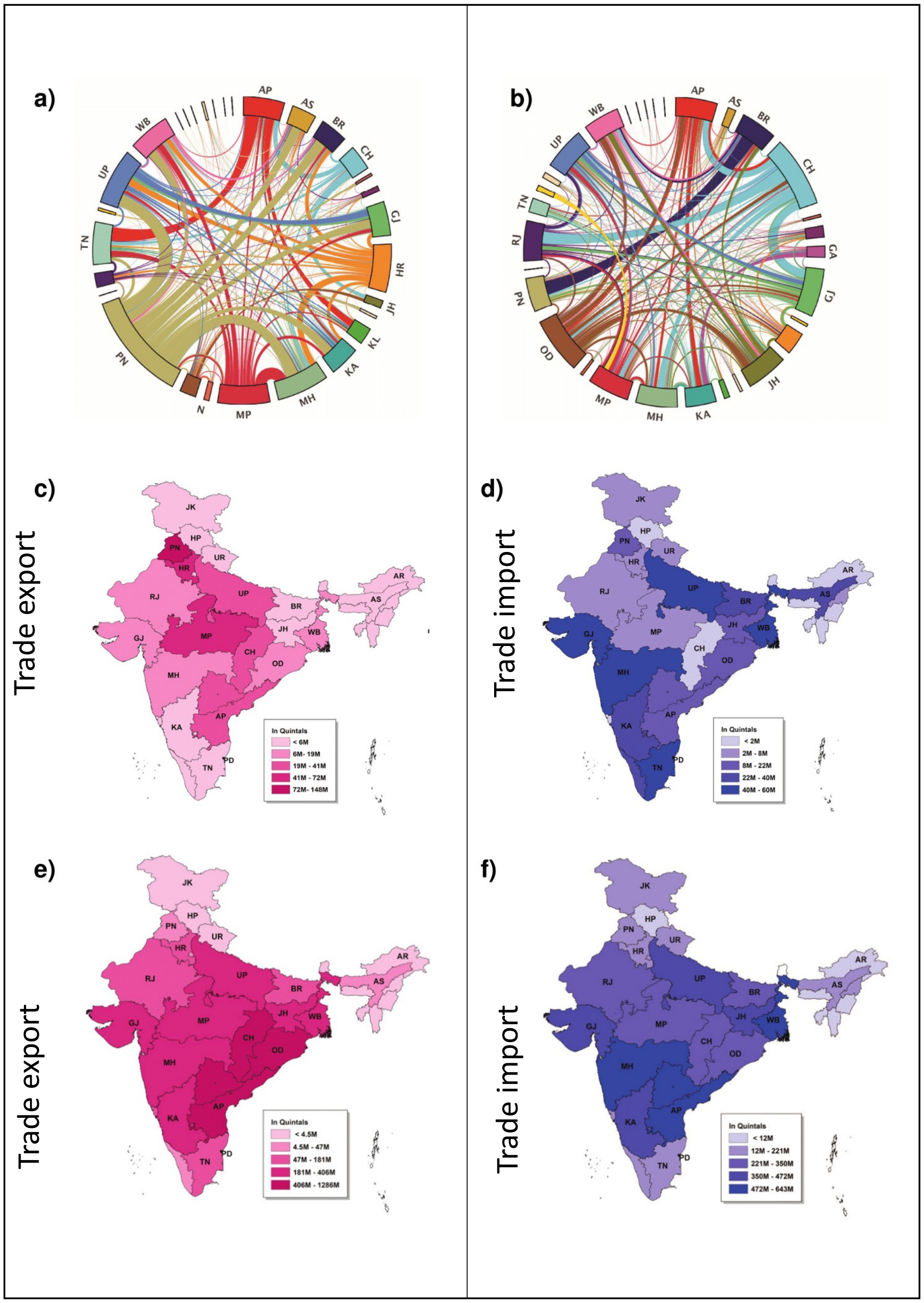

Fig 1. Interstate trade network based on physical values (weights). The chord diagrams show the average trade flows between different states in India. The links' width showcases the trade volume in quintals, and the links' colours correspond to the exporting regions (a and b), depicting the trade network for Agricultural and Non-Agricultural commodities. (c and d) shows average exports and imports for Agriculture and (e)and (f) for Non-agriculture commodities in the study area.

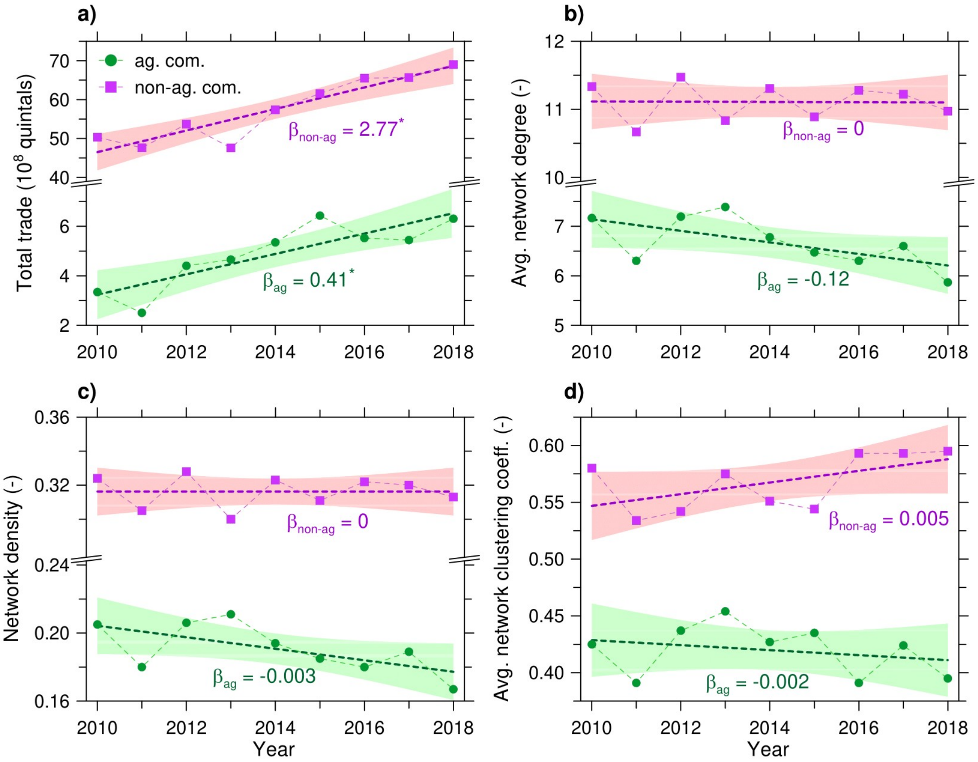

Fig 2. Temporal changes of total trade volume and network characteristics. a) Total traded value(in 10^8 quintals), (b) Average degree, (c) Network density, and (d) Average Clustering Coefficient for 2010-2018.here, β indicates the slope of a linear trend line fitted to scattered points (values with * represent significant trend at p-value < 0.05). The results are presented for the Agriculture (green) and Non-Agriculture (purple) interstate trade networks.

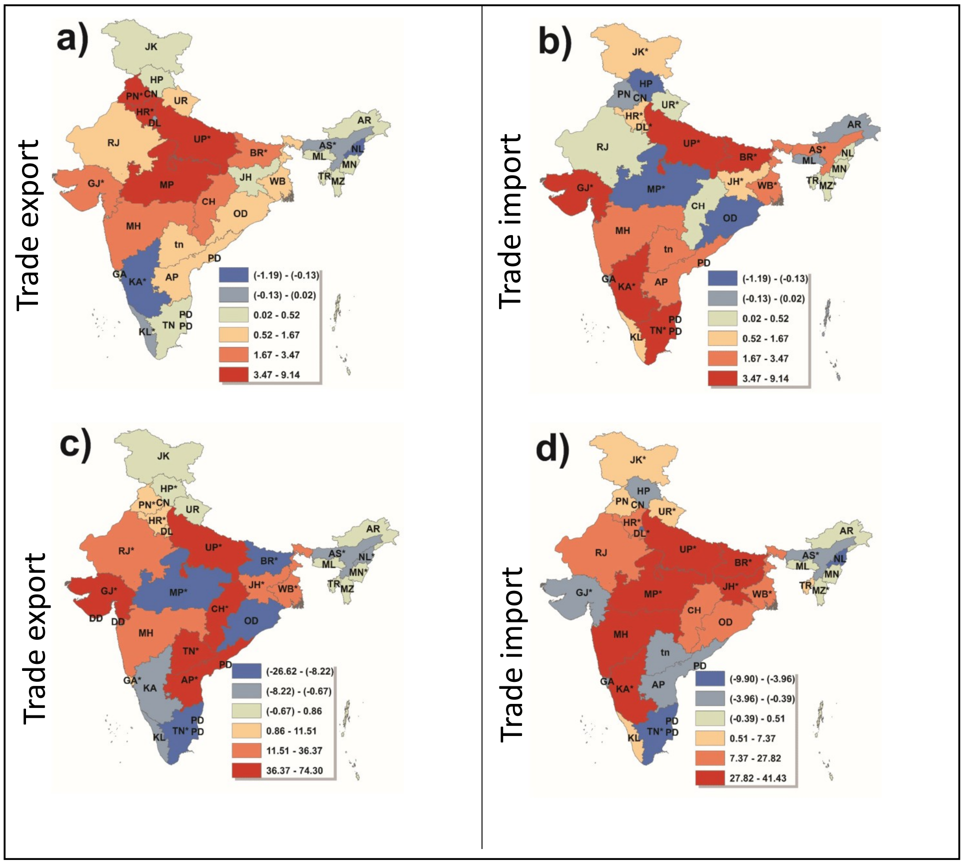

Fig 3. Spatial variation in trends of trade import and trade export a) the spatial variation in the trend of exports and imports of Agriculture (a and b) and Non-agriculture (c and d)commodities over the period of 2010-2018. All values are in Million Quintals.

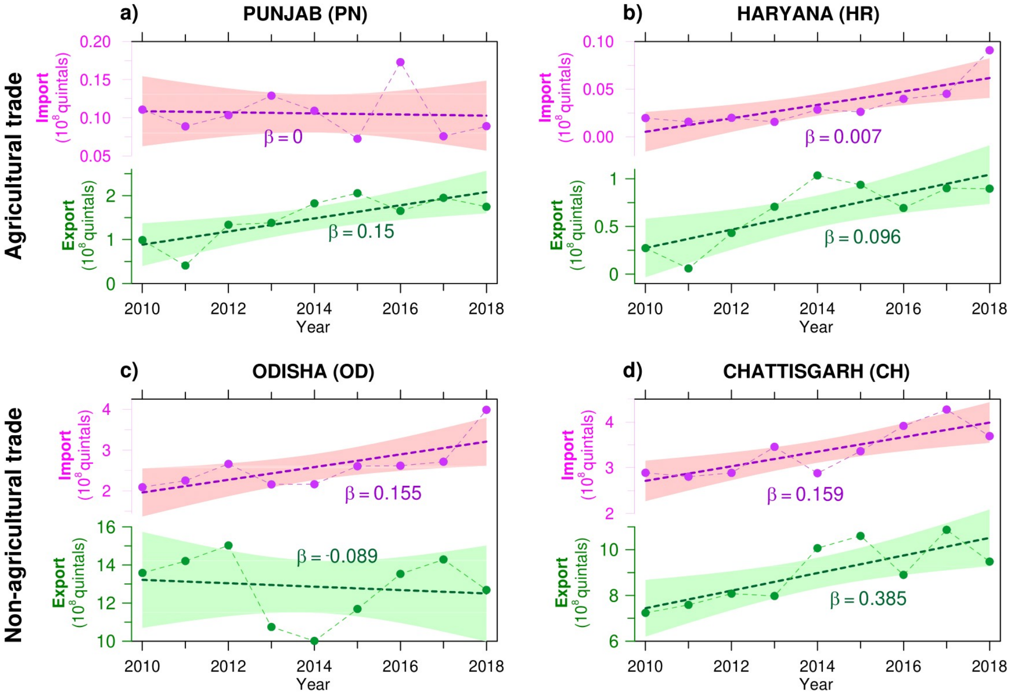

Fig 4. Temporal variation of exports and imports of leading exporters The temporal variation of exports and imports of leading exporters of agriculture (Punjab and Haryana) and non-agriculture (Odisha and Chhattisgarh) commodities over the period 2010–2018. Also shown are the respective linear regression slope (β).

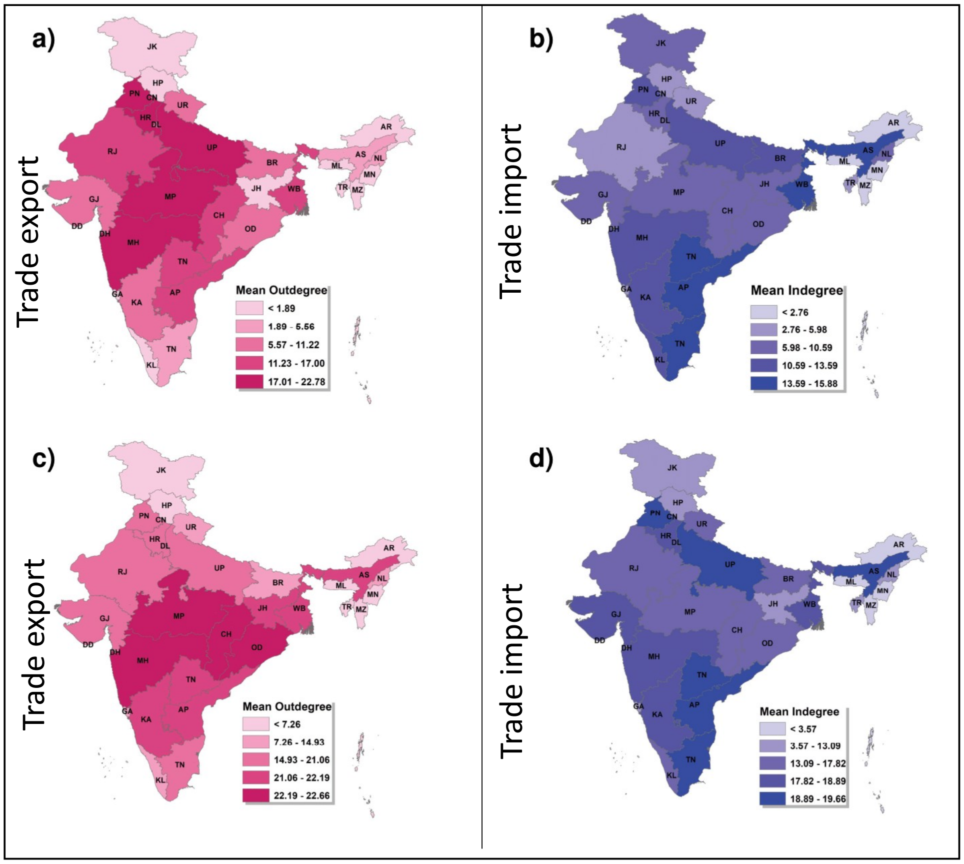

Fig 5. Spatial and temporal variations of indegree and outdegree Study area showing average outdegree and average indegree respectively for agriculture (a and b) and non-agriculture (c and d) commodities. All averages are calculated over the period of 2010-2018.

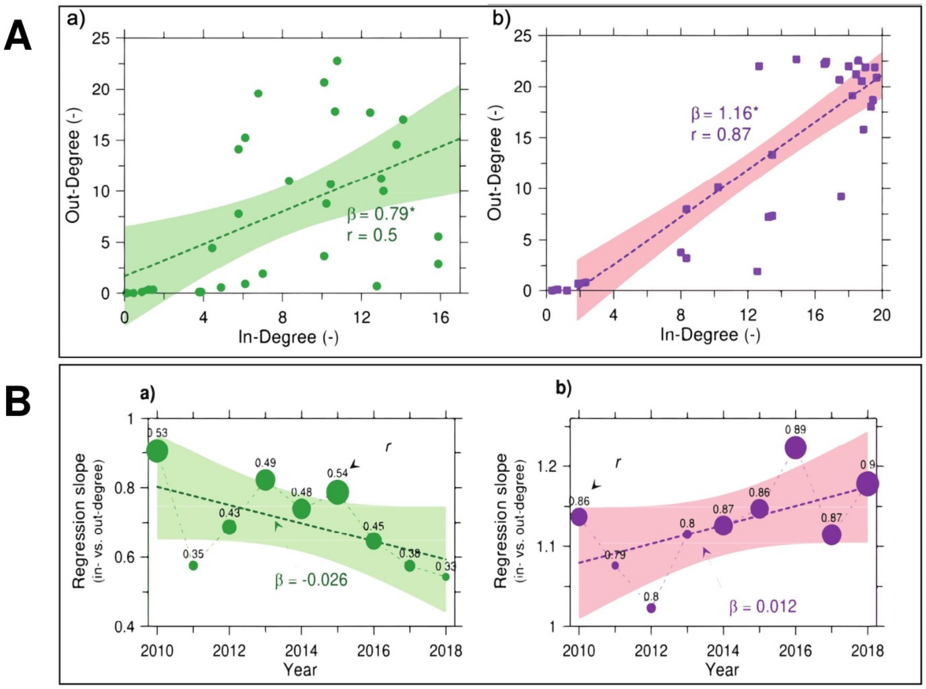

Fig 6. Statistical relationship between indegree and outdegree (A) Scatter plots of average indegree and outdegree over nine years (2010-2018) for Indian states. β indicates the slope of the fitted linear regression line to scattered points, and r indicates the Pearson correlation coefficient. (B) Scatter plots of the year-wise slope of indegree and outdegree. The size and numerical above the marker represent the respective correlation values. In all panels, shown are the fitted regression line (dash line) and corresponding 95 % confidence band. The results in sub-panels (a) and (b) are for agriculture trade and non-agriculture trade, respectively.

Table 1. The monetary value of each commodity in the window of 2010-2018

**Commodity**	**Monetary value (₹per quintal)**								
	2010	2011	2012	2013	2014	2015	2016	2017	2018
Cement	486	474	474	474	474	474	448	462	494
Coal and coke	427.64	427.64	427.64	427.64	741.67	849.66	786.84	870.11	1098.63
Coffee	10564.6	11254.1	14041.3	15211.9	15511.3	17814.6	16310.9	15840.3	15720.5
Fertilizers	1364.26	1364.26	1478.07	1699.73	1866.22	1968.77	2035.74	1920.72	1662.99
Gram and Gram products	1760	2100	2800	3000	3100	3175	3500	4000	4400
Iron and Steel	2623.58	2809.04	3211.36	3461.63	3455.45	3490.45	3222.98	3024.21	3587.68
Iron ore	370.70	370.70	386.75	422.40	376.38	376.36	358.52	340.68	322.83
Jowar and Bajra	850	890	990	1347.5	1385	1400	1432.5	1490	1575
Jute	1375	1575	1675	2200	2300	2400	2700	3200	3500
Kerosene	1218	1218	1439.75	1448.5	1448.5	1495	1498.25	1498.25	2004
Limestone, dolomite and Gypsum	32.89	35.71	40.25	45.71	46.23	49.75	48.77	55.80	57.37
Maize and millets	877.5	922.5	1015	1337.5	1405	1430	1487.5	1545	1662.5
Manganese ore	759.89	946.79	897.82	996.97	1123.95	1127.37	856.47	914.46	1511.73
Marble stones	1105.07	626.44	1187.69	1463.61	1686.39	1837.42	2068.97	2155.96	2157.75
Oil seeds	2798.96	2648.30	3258.86	4733.53	48623.37	4586.57	4713.47	4380.30	3816.74
Pulses	2362.5	2830	3200	3862.5	4012.5	4093.75	4375	4806.25	5168.75
Rice	965	1015	1095	1265	1327.5	1380	1430	1490	1570
Spices	15776.24	16543.82	16971.26	17243.64	16806.84	16667.80	19256.61	18637.68	17440.18
Sugar	2463.34	3156.40	3188.58	3069.79	2897.31	2725.48	2555.51	3403.89	2972.59
Vegetable and Fruits	1774.16	1941.14	2007.59	2051.47	3058.66	3051.46	3470.25	2554.30	3301.35
Wheat	1100	1120	1285	1350	1400	1450	1525	1625	1735

Table 2. Changes in the trade network connection from 2010 to 2018

Year	Agricultural Trade		Non - Agricultural Trade	
	Nodes	Links	Nodes	Links
2010	26	210	28	325
2011	28	193	28	294
2012	28	219	26	323
2013	28	218	27	323
2014	27	206	25	295
2015	27	198	26	294
2016	26	182	27	281
2017	27	200	29	299
2018	27	181	30	303

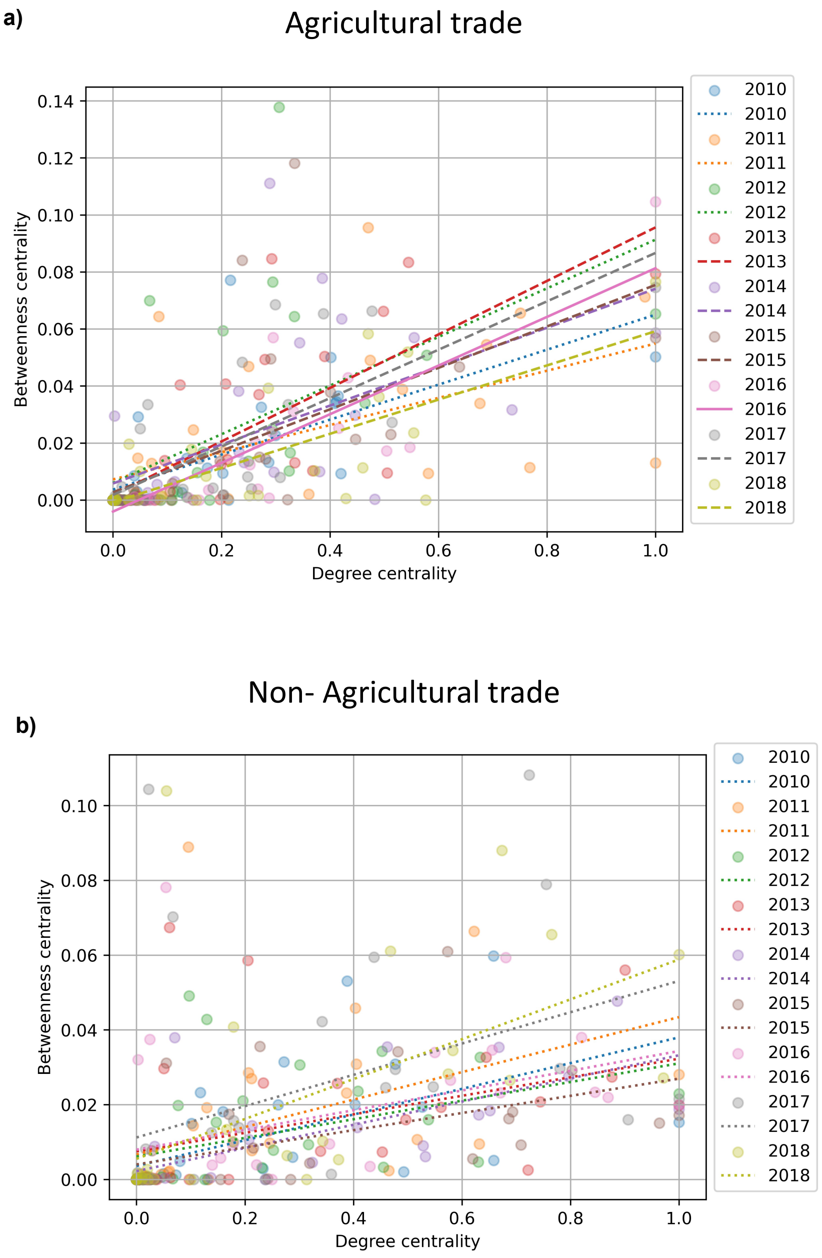

Fig 7. Spatio-temporal network centralization metrics. (a and b) shows each state's betweenness and degree centrality over the temporal window of 2010 to 2018 for agricultural trade and non-agricultural trade, respectively. The solid line represents the correlation coefficient r > 0.6, the dashed line depicts the r between 0.5 and 0.6, whereas the dotted line represents r< 0.5

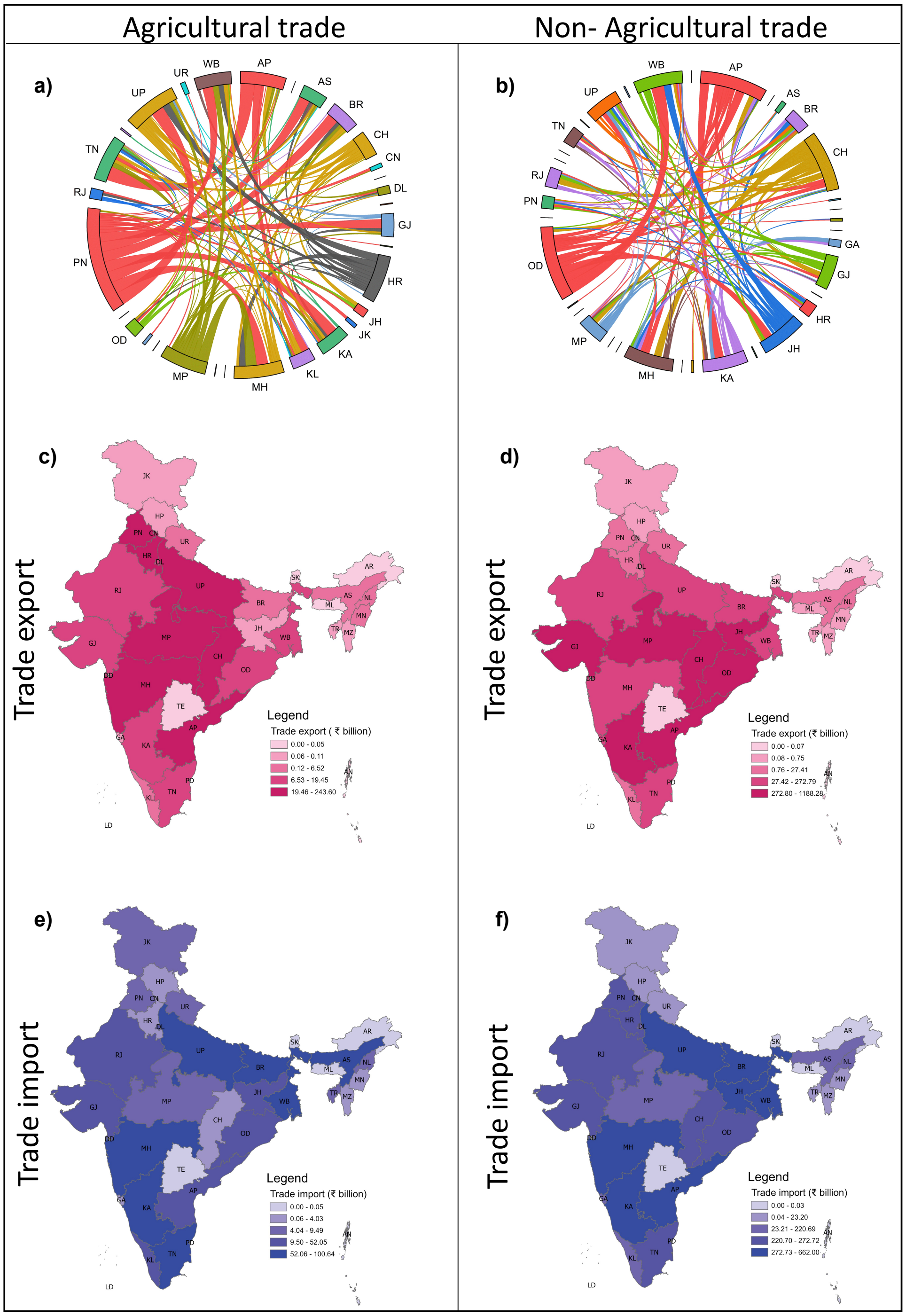

Fig 8. Interstate trade with inflation-adjusted values of agriculture and non-agriculture commodities in India. (a) and (b) show the chord diagram representing the time-averaged agricultural exports and non-agricultural commodities, respectively. The link indicates the flow between different states, the link width showcases the trade volume in inflation-adjusted |, and the links' color corresponds to the exporting regions. (c) and (e) shows time-averaged exports and imports for agriculture, whereas (d) and (f) depict the same for non-agriculture commodities. The state is represented by two letters. All averages are calculated for the period 2010-2018.

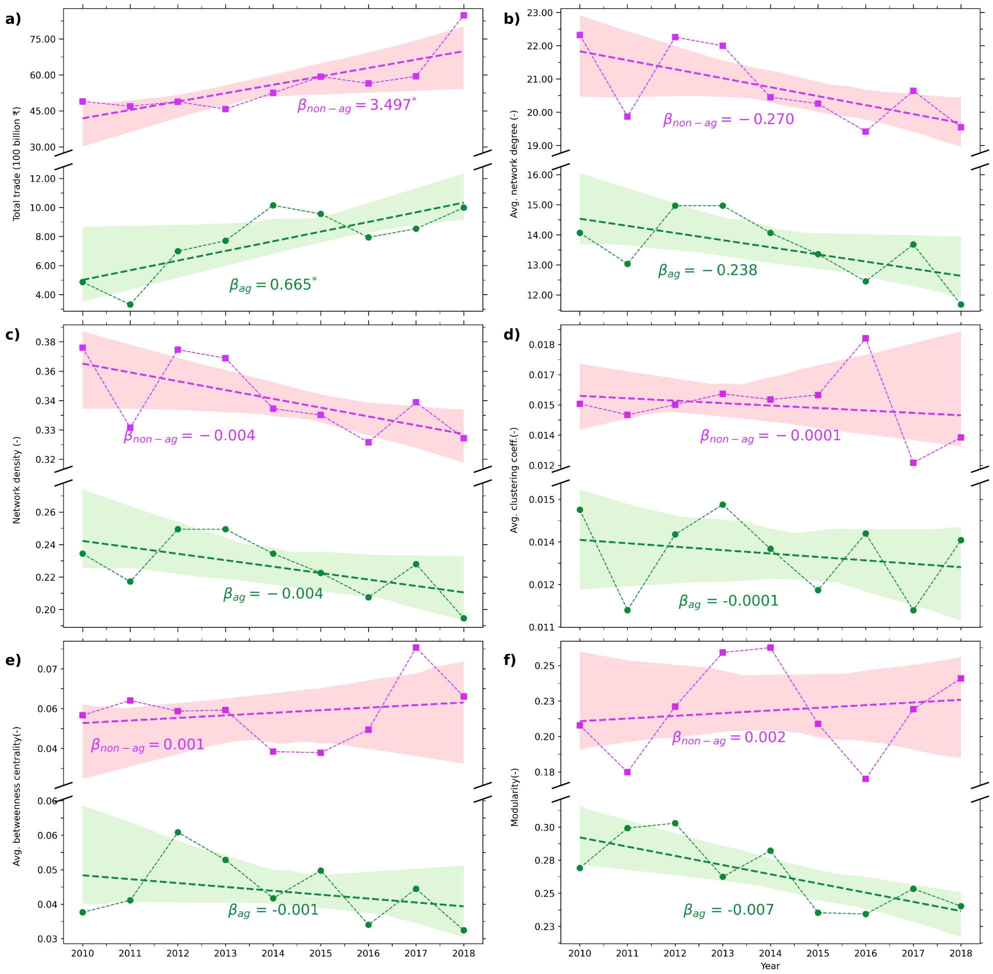

Fig 9. Temporal changes in total trade value and topological characteristics of the network based on inflation-adjusted values. (a)Total traded value (in inflation-adjusted 100 billion ₹), (b) Average network degree, (c) Network density, (d) Average clustering coefficient, (e) Average betweenness centrality, and (f) modularity for 2010-2018. here, β indicates the slope of a linear trend line fitted to scattered points (values with * represent a significant trend at $p - value < 0.05$). The results are presented for the Agriculture (green) and Non-Agriculture (purple) interstate trade networks.

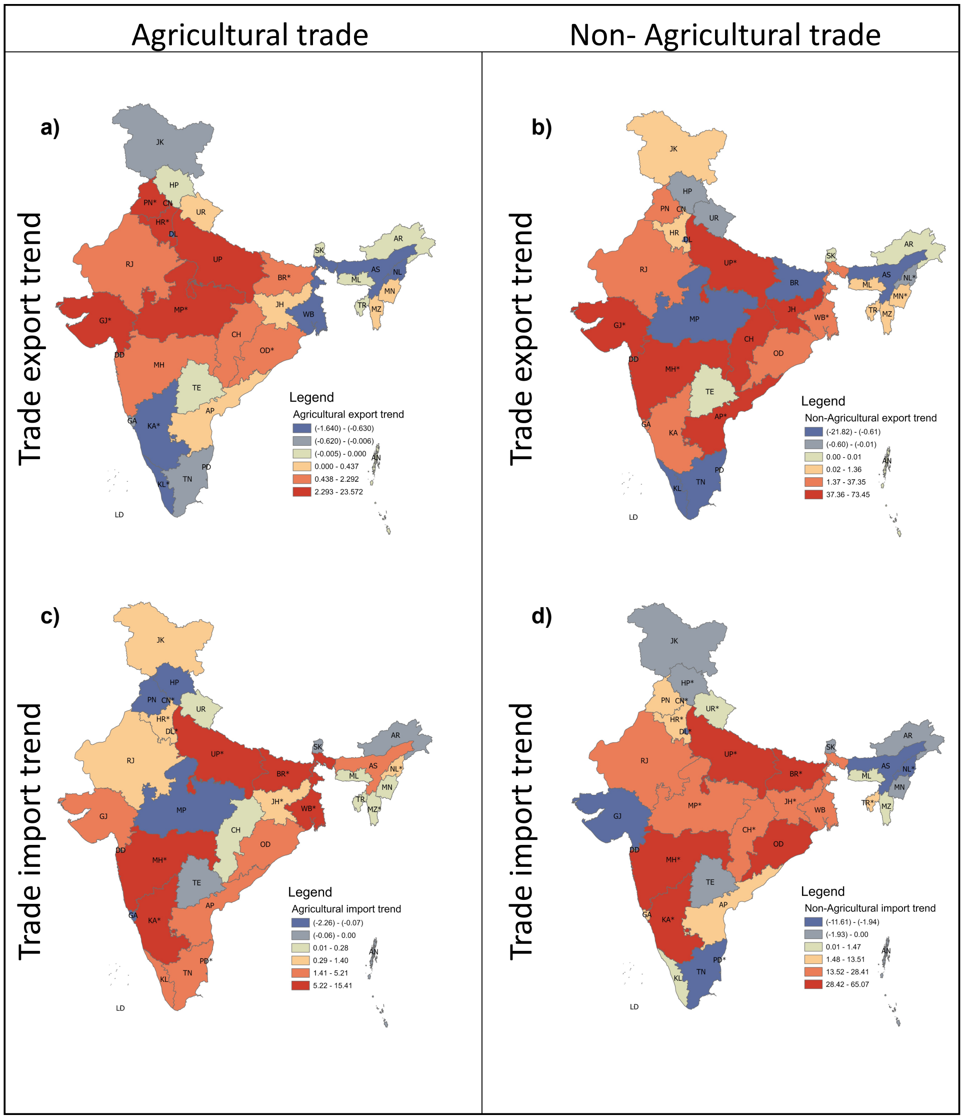

Fig 10. Spatial variation in the trends of exports and imports. (a and c) shows the spatial variations of export and import for agricultural products through trade trends for inflation-adjusted values, whereas (b) and (d) present the same for Non-agriculture commodities. Here the states with * represent states with a significant trend (at p-value < 0.05) of commodities over the period of 2010-2018. All values are in inflation-adjusted billion ₹.

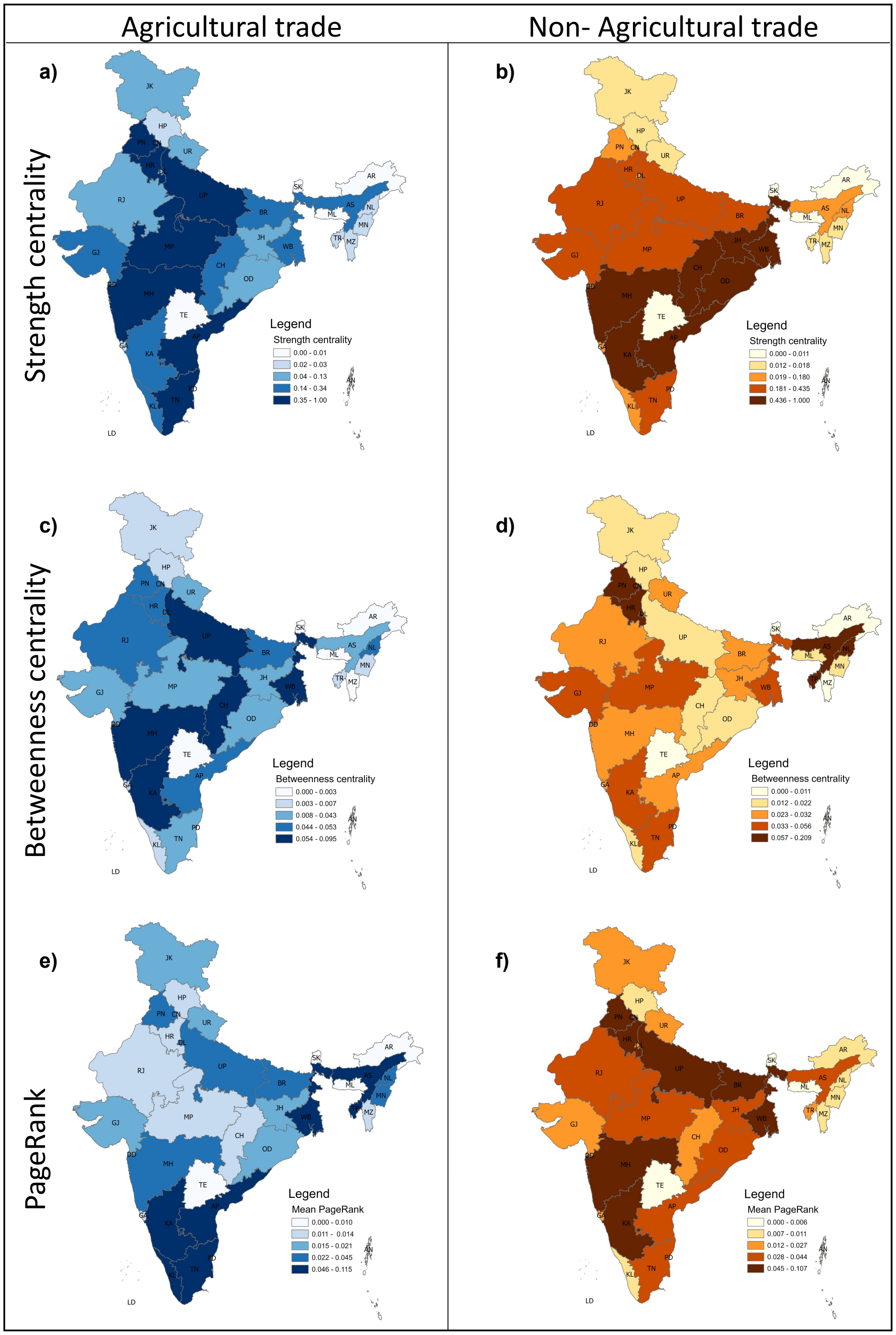

Fig 11. Relative influence of states on interstate trade network with inflation-adjusted monetary values. (a) and (b) shows the time-averaged strength centrality of each state for agriculture and non-agriculture DITN, respectively, (c) and (d) depict the time-averaged betweenness centrality for the same. In comparison, (e) and (f) indicate the time-averaged PageRank centrality of each state.

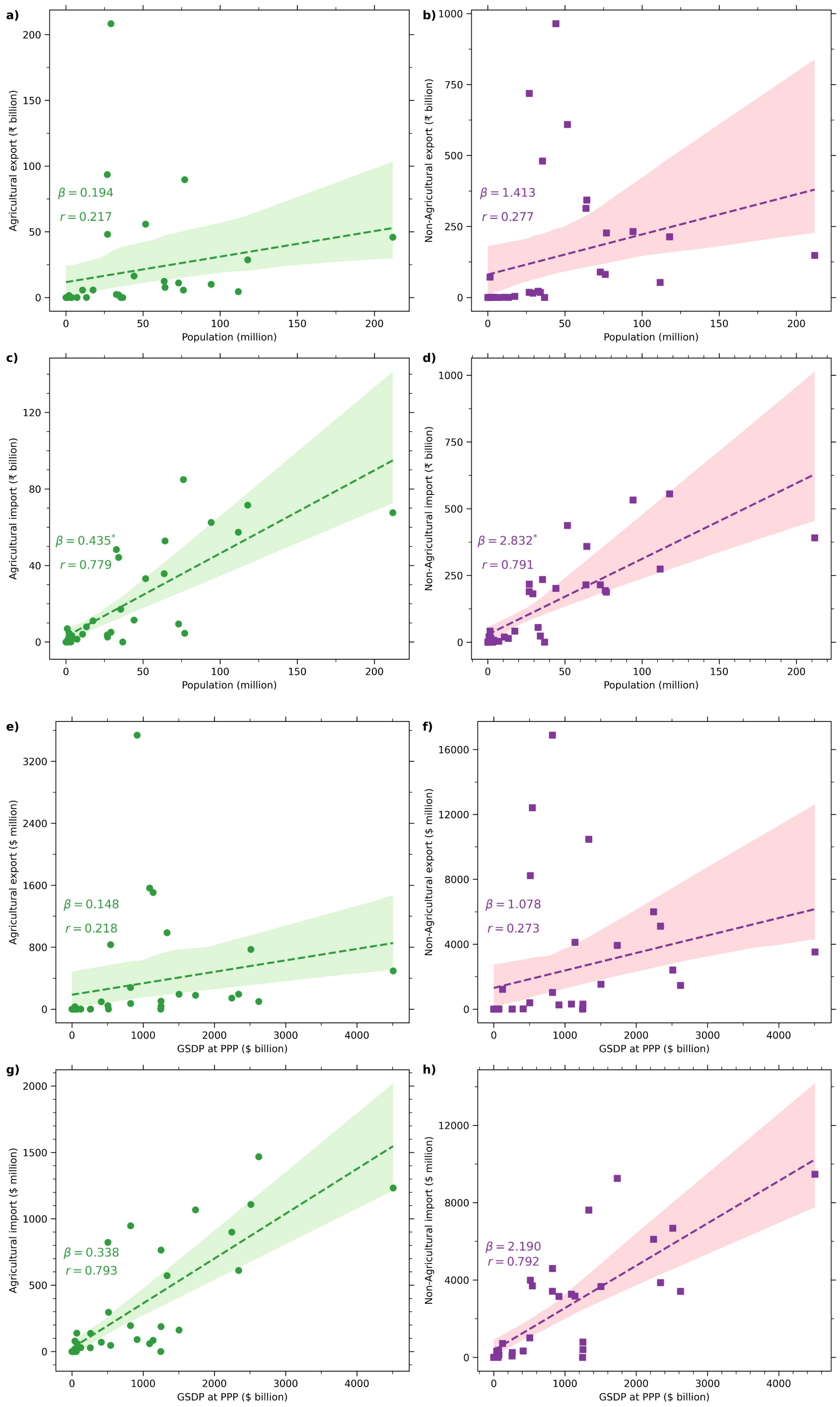

Fig 12. Statistical relationship between trade export/import and population/GSDP at PPP. (a) and (c) shows the statistical relationship between the time-averaged population vs export and import of each state for agriculture, whereas similarly (b) and (d) show the same for non-agriculture DITN, respectively. (e) and (g) depict the statistical relationship between time-averaged GSDP at PPP and the export/import of each state for agriculture. In comparison, (f) and (h) show the same for non-agriculture DITN.

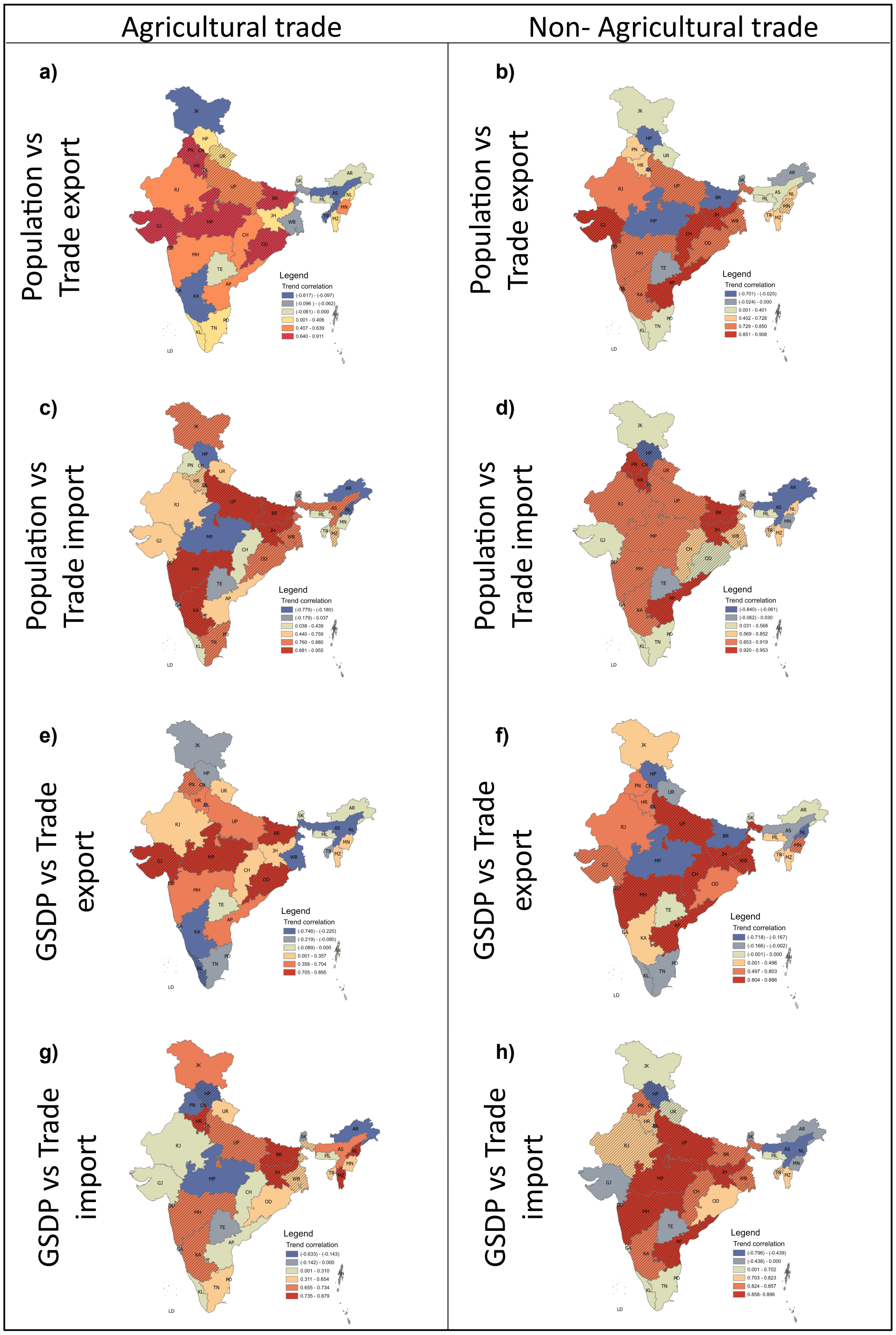

Fig 13. Statistical relationship between trade export/import trend and population/GSDP at PPP trend. (a) and (c) shows the statistical relationship between the population trend vs the export and import trend of each state for agriculture, whereas (b) and (d) shows the same for non-agriculture DITN, respectively. (e) and (g) depict the statistical relationship between GSDP at PPP trend and each state's export/import trend for agriculture. In comparison, (f) and (h) show the same for non-agriculture DITN. The hatched portion represents the significant correlation between trends.
